# Catfish Transgenesis and Gene Editing: Overview, Recent Advances, and Future Perspectives

**DOI:** 10.3390/biology15161365

**Published:** 2026-08-11

**Authors:** Ahmed H. Elaswad, Karim M. Khalil, Rex A. Dunham

**Affiliations:** 1 Center of Excellence in Marine Biotechnology, Sultan Qaboos University, Muscat 123, Oman; 2 Department of Animal and Veterinary Sciences, College of Agricultural and Marine Sciences, Sultan Qaboos University, Muscat 123, Oman; 3 School of Fisheries, Aquaculture and Aquatic Sciences, Auburn University, Auburn, AL 36849, USA

**Keywords:** catfish, CRISPR/Cas9, gene editing, genome editing, transgenic fish

## Abstract

Transgenesis and gene editing offer a promising approach for more efficient and sustainable aquaculture by generating high-performance fish to enhance global food security. This article provides a comprehensive review of the history, current status, and future perspectives of employing transgenesis and gene-editing technologies to improve fish production and welfare, focusing on catfishes, which represent a diverse group of economically important fish. We discussed the application of various genetic technologies, including gene transfer (transgenesis) and gene editing, to boost fish economic traits and achieve genetic confinement. We also reviewed the progress on integrating transgenesis and gene-editing techniques for multiple gene editing, an approach that can speed up and make the genetic modification of fish more efficient. This review also highlights several research gaps in the current literature on gene transfer and gene editing in catfishes. Specifically, it provides directions for future catfish research, including the employment of transcriptomics in phenotypic assessment of transgenic and gene-edited fish, addressing compensatory mechanisms when selecting the gene-editing targets, and accounting for gene-environment and gene-nutrition interactions. It also highlights the application of advanced gene-editing tools, integration of genomic selection and artificial intelligence with gene editing, transparent public education about the benefits and risks of genetic biotechnology, and the development of effective reproductive confinement strategies.

## Simple Summary

This review offers in-depth insights into catfish transgenesis and gene editing, starting from the first attempts to generate transgenic fish, going through the significant progress made in improving catfish productive traits, and comparing the effectiveness of different tools such as traditional gene transfer, CRISPR-assisted gene insertion, and gene knockout. The review also discusses the potential risks and ecological concerns for transgenic and gene-edited fish. Finally, this review highlights the limitations of current research on catfish transgenesis and gene editing and discusses recommendations for future research.

## 1. Introduction

Although fish comprise the most diverse group of vertebrates, with an estimated number of 30,000 species, a much smaller number of fish species are used for research and commercial purposes. According to the latest estimate, the number of aquatic animal species entering domestication, already 430, is 10-fold greater than that of all domesticated land animal species, which has now plateaued at 44 [1]. The major cultured species worldwide include salmonids, catfishes, tilapias, carps, shrimps, and Mollusca. Currently, there are approximately 36 extant catfish families with more than 3000 species described [2,3]. This makes the catfishes (Order: Siluriformes) the second most diverse vertebrate order. In fact, one out of every 20 vertebrate species is a catfish. Three catfish families, Ictaluridae, Clariidae, and Pangasiidae, contain important species used in aquaculture worldwide. These species include the channel catfish (*Ictalurus punctatus*), blue catfish (*Ictalurus furcatus*), African catfish (*Clarias gariepinus*), and striped catfish (*Pangasianodon hypophthalmus*).

In the past, the channel catfish was the primary species used in the aquaculture industry in the United States due to its superior production traits. Recently, the hybrid from crossing the channel catfish female with the blue catfish male (CB) accounts for 70% of US catfish production [4,5]. Compared with the parental species, the CB hybrid catfish exhibited superior traits, including higher growth rates, resistance to bacterial and parasitic diseases, tolerance to low dissolved oxygen levels, better carcass yield, and vulnerability to angling [6,7,8,9,10,11,12,13,14,15]. After two decades of steady expansion from the 1980s to the 2000s, the catfish industry experienced a sharp decline after its 2003 peak of 300 million kg. Production subsequently decreased by over 50%, falling to 127 million kg and 138 million kg in 2008 and 2011, respectively, before ultimately stabilizing at approximately 150 million kg [16,17,18]. The CB hybrid catfish is a key player in the recovery of the American catfish industry due to its superior performance [5]. In aquaculture, intensive systems, such as in-pond raceways [19] and split ponds [20], can double the production but will work only with CB hybrid catfish. The growth and disease resistance of channel catfish and blue catfish usually decline in these intensive systems. Therefore, harnessing the power of biotechnology in generating fast-growing disease-resistant strains and overcoming the reproductive barriers between channel catfish and blue catfish would increase catfish production and profitability.

Beyond North American catfish, the Pangasiidae family, especially *P. hypophthalmus* (Pangasius or Basa), is globally significant because of its highly intensive production. The Mekong Delta in Vietnam is the global epicenter, producing 1.525 million tons of *P. hypophthalmus* in 2022, with exports to over 138 countries valued at US $1.6 billion [21]. Production is highly intensive, with hatcheries annually generating billions of larvae. The Clariidae family, often referred to as African and Asian catfishes and best known by the species *C. gariepinus*, holds significant economic and social importance across Africa and Asia. Leading producers, including Nigeria, Kenya, and Indonesia, highly value these fish due to their remarkable adaptability, rapid growth, and resilience to diverse and sometimes challenging farming conditions [22,23,24]. Production techniques vary widely, ranging from high-volume, traditional earthen ponds in resource-rich regions to sophisticated, modern systems like recirculating aquaculture systems (RASs), biofloc, and aquaponics, which are used to enhance efficiency and sustainability in countries like Indonesia and some parts of Europe. While these catfishes are vital for rural livelihoods and food security, the industry of both families faces challenges in disease management, feed costs, and sustainability.

To address these shared challenges, recent rapid advancements in molecular biology have specifically enabled the development of transgenic and gene-edited animals. A good example of a transgenic fish is the growth hormone-transgenic Atlantic salmon (*Salmo salar*), called AquAdvantage salmon, which was approved for human consumption by the Food and Drug Administration (FDA) on 19 November 2015 [25]. The AquAdvantage salmon was produced in two land-based FDA-approved facilities: one for broodstock in Canada, and the other for raising market fish from eggs in Indiana, USA. This is the first transgenic animal commercially approved for human consumption in the United States and may not be the last. In AquAdvantage salmon, the growth hormone coding sequence from Chinook salmon (*Oncorhynchus tshawytscha*) driven by the antifreeze protein gene promoter from ocean pout (*Zoarces americanus*) was transferred into the AquAdvantage salmon genome, resulting in significant growth enhancement. Unfortunately, on 11 December 2024, AquaBounty announced plans to cease fish farming operations due to insufficient funds [26].

In catfish, the genetic approaches offer great potential to improve production, including gene transfer and gene editing, to generate fast-growing, disease-resistant strains. Therefore, this review provides a critical analysis of gene transfer and genome editing strategies aimed at enhancing catfish production. Moving beyond a foundational overview, we discuss the history, recent advances, opportunities, challenges, limitations, research gaps, and future directions of catfish transgenesis and gene editing. The primary objective of this work is to evaluate how these technologies can be tailored to overcome the distinct physiological challenges and production goals of this globally significant aquaculture group. Furthermore, we explore the critical role of transgenic sterilization as a regulatory and environmental prerequisite for the commercialization of transgenic lineages and whether genetically modified catfishes will be approved for human consumption in the future.

## 2. Literature Search Methodology

The literature search was conducted mainly through Scopus and Google Scholar databases using the following search keywords: catfish biotechnology, transgenic catfish, gene transfer catfish, gene editing catfish, CRISPR catfish, gene knockout catfish, genome editing catfish, transgenesis pleiotropic effects, gene editing pleiotropic effects, catfish reproductive confinement, catfish sterilization, and ecological concerns catfish biotechnology. Reference lists of relevant articles were also examined to identify publications related to this review. During the search, no geographical restrictions were applied; studies from all countries were considered when relevant. To cover the history and recent advances in catfish transgenesis and gene editing, the search was conducted with the earliest available research and was updated until July 2026. Eligible sources were selected based on their relevance to the study, including peer-reviewed journal articles, books, book chapters, and conference proceedings. A few official websites and technical reports from recognized governmental and international organizations were consulted to obtain information on catfish production and the status of transgenic fish. Graduate theses were used only when the relevant information was not published in a peer-reviewed journal. Conference abstracts and unpublished data were excluded.

## 3. Progress on Catfish Transgenesis and Gene Editing

A common approach in modern fish genetics research is manipulating the genes (genotype) of embryos and observing the impact of this change on the organism’s performance (phenotype). This genetic manipulation comprises several techniques in which genes can be transferred (transgenesis), knocked down (gene silencing), permanently inactivated (gene knockout), or rewritten (gene editing). These techniques were applied to fish to generate new strains with enhanced performance, including growth enhancement [27], disease resistance [28,29], cold tolerance [30], generation of novel ornamental fish [31], fish pharming (production of pharmacological proteins using fish as a biofactory) [32], monitoring environmental pollution [33], enhancing nutritional value of fish meat [34], transgenic sterilization [35,36], functional genomics, and developmental biology [37]. Gene editing tools were also utilized to generate fast-growing [38,39,40,41,42], disease-resistant [43,44], and omega-3-rich fish [45,46].

## 4. Transgenesis

Transgenesis is the insertion of an exogenous DNA—called a transgene—into sperm, unfertilized eggs, fertilized eggs, or gonadal cells of a living organism so that the transgene is integrated into the organism’s genome and transmitted to its offspring. Attempts to produce transgenic fish began in the mid-1980s, just after the generation of the first transgenic animal [47]. Zhu et al. [48] introduced the human growth hormone gene driven by the metallothionein promoter into goldfish (*Carassius auratus*). Since then, gene transfer has been reported in several fish species, such as rainbow trout (*Oncorhynchus mykiss*) [49], mud loach (*Misgurnus anguillicaudatus*) [50], channel catfish [51,52], Atlantic salmon [27,53], zebrafish (*Danio rerio*) [52,54], Japanese medaka (*Oryzias latipes*) [55], common carp (*Cyprinus carpio*) [52,56,57], Nile tilapia (*Oreochromis niloticus*) [58,59], northern pike (*Esox lucius*) [60], coho salmon (*Oncorhynchus kisutch*), rainbow trout, cutthroat trout (*Oncorhynchus clarkii*), Chinook salmon [61], Arctic char (*Salvelinus alpinus*) [62], and mud loach (*Misgurnus mizolepis*) [63]. In this section, we will focus on the attempts to generate transgenic catfish (Table 1).

### 4.1. Transgenesis for Growth Enhancement

In 1986, the first transgenic fish (growth hormone-transgenic channel catfish) in the United States and the fourth worldwide was generated at the Fish Genetics Research Unit, E. W. Shell Fisheries Center, Auburn University [51]. Following this initial success, most of the transgenic catfish research focused on three main areas: growth enhancement, disease resistance, and transgenic sterilization. Additionally, during this period, Auburn University established outdoor confinement facilities for transgenic fish research and obtained government approval for the first outdoor research on a transgenic animal worldwide, enabling Auburn University to lead transgenic catfish research.

Focusing first on growth enhancement, growth hormone-transgenic fish of several teleost species were typically shown to grow 2–11-fold faster than non-transgenic controls. The great potential of transgenic technology was demonstrated in transgenic coho salmon, which exhibited an average 10-fold increase in growth compared to non-transgenic controls, with the largest fish reaching more than a 30-fold increase in body weight [61]. In mud loach, transferring the mud loach growth hormone gene driven by the mud loach β-actin promoter resulted in up to 35-fold improvement in growth, a rate that probably can never be achieved by traditional breeding. Other growth hormone-transgenic fish species exhibited faster growth rates, such as Nile tilapia [80], common carp [56], rainbow trout [61,81], cutthroat trout, coho salmon, and Chinook salmon [61].

In channel catfish, growth hormone transgenic individuals were first generated by microinjecting one-cell embryos with the human growth hormone gene driven by the mouse metallothionein promoter [51]. Then, two salmonid growth hormone genes from rainbow trout and coho salmon, driven by Rous Sarcoma Virus Long Terminal Repeat (RSVLTR-rtGH and RSVLTR-csGH), were microinjected into channel catfish embryos. In two F_1_ families transgenic for RSVLTR-csGH, the growth rate was improved by 26% compared with non-transgenic full siblings [65]. Recently, channel catfish fertilized eggs were electroporated with two transgene constructs containing the channel catfish growth hormone cDNA (ccGH) driven by either the ocean pout antifreeze protein promoter (opAFP) or the rainbow trout metallothionein promoter (rtMT) to generate growth hormone transgenic channel catfish, which exhibited larger body weights in different rearing systems. In a flow-through system, sixteen-week-old opAFP-ccGH F_1_ transgenic fish demonstrated a 1.41–1.62-fold increase in growth compared with control fish, similar to rtMT-ccGH F_1_ transgenic fish, which grew 1.23–1.78 times faster than control fish [82]. In earthen ponds, the difference in body weight between growth hormone-transgenic fish and non-transgenic full siblings was magnified as the fish grew. In 48-month-old F_2_ fish, for example, F_2_ opAFP-ccGH and F_2_ rtMT-ccGH transgenic channel catfish were 2.58- and 2.99-fold larger than their non-transgenic siblings, respectively [83].

It is important to note that the constitutive overexpression of growth hormone (GH) in transgenic fish fundamentally alters the GH-Insulin-like Growth Factor (IGF) axis, which is the primary regulatory pathway for somatic growth, involving components such as the growth hormone receptors (*ghr1*, *ghr2*) and the insulin-like growth factors (*igf1*, *igf2*). Notably, research in transgenic catfish demonstrated a significant reversal of sexual dimorphism. While non-transgenic males are typically larger, transgenic females exhibited significantly higher body weights than males by 48 months of age [83]. This outcome, which is also observed in growth hormone-transgenic tilapia and coho salmon, strongly suggests that this transgene may differentially interact with or modify the hormonal pathways of females post-maturation, leading to a disproportionate enhancement of growth in that sex [84,85,86].

**Figure 1:**
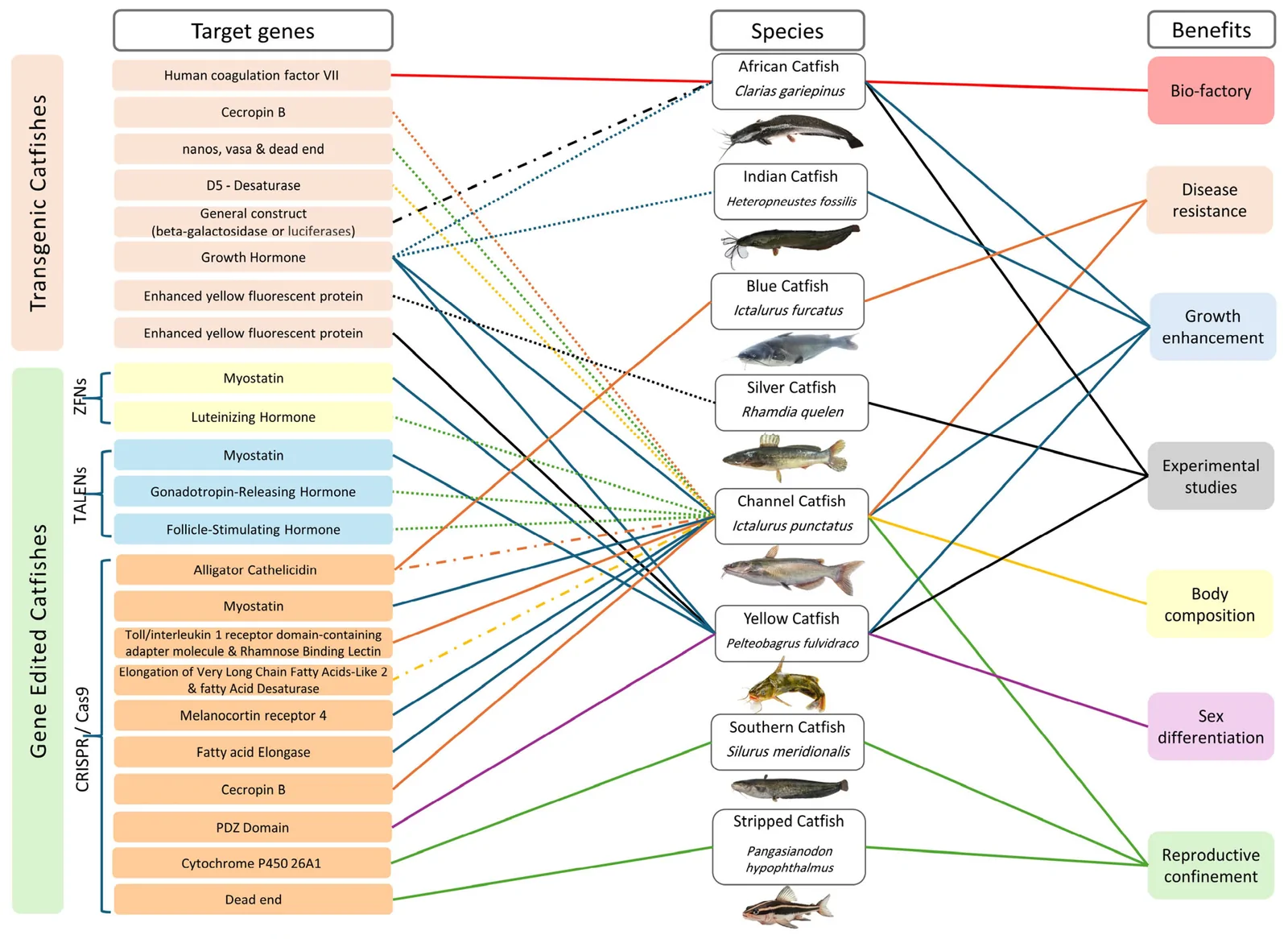
Applications of transgenesis and gene-editing techniques in various catfish species. The left panel illustrates representative target genes associated with key biological functions applied in both transgenic and gene-editing approaches (ZFNs, TALENs, and CRISPR/Cas9). The lines connect the association between specific target genes and the respective catfish species in which they have been investigated or modified (solid line: microinjection; small dashes: electroporation; interrupted line: microinjection & electroporation). The right panel summarizes the major phenotypic and applied outcomes achieved through these approaches. The colored connectors linking species to outcomes denote experimentally validated or proposed trait improvements resulting from targeted genetic manipulation.

Other growth hormone transgenic catfish species were also generated. An additional copy of the *C. gariepinus* growth hormone gene was transferred to Mutiara catfish (*C. gariepinus*), and the growth rate was improved in the second, third, and fourth generations. The second generation of these transgenic fish demonstrated a 150–200% increase in growth compared with non-transgenic fish [87]. The one-month-old third-generation fingerlings displayed a 2–3-fold improvement in growth relative to control fish, particularly when supplemented with balanced diets [88]. In the fourth generation, growth hormone-transgenic Mutiara fish exhibited a higher expression level of growth hormone (1.3–2.9 fold) and larger body weight (1.9–2.3 fold) than non-transgenic control fish at higher stocking densities [89]. Interestingly, the fifth-generation females exhibited higher levels of growth hormone and estradiol, with larger ovaries, compared with non-transgenic fish [75]. Overall, these studies demonstrate the remarkable capacity of growth hormone transgenesis to accelerate growth performance in catfish and other teleosts beyond the limits achievable through conventional selective breeding. Figure 1 shows the progress of transgenic and gene-editing techniques and their applications in catfish species.

### 4.2. Transgenesis for Disease Resistance

Disease resistance is an economically important trait in aquaculture. Gene transfer has also been undertaken to enhance resistance to pathogens in several fish species. Several genes were transferred to boost disease resistance in fish, including cecropin [90], lactoferrin [91], hepcidin [92], epinecidin-1 [93], and lysozyme [94]. In addition to providing long-term protection, transgenes can be inherited by future generations, protecting fish during early life stages, especially when the immune system is not fully developed.

Cecropins are antimicrobial peptides initially identified in the hemolymph of the silkmoth *Hyalophora cecropia*. Cecropins are effective against several pathogens, including bacteria, viruses, fungi, and protozoa (reviewed in Elaswad and Dunham [95]). The resistance to *Pseudomonas fluorescens* and *Vibrio anguillarum* was enhanced in cecropin transgenic medaka [90]. In channel catfish, the cecropin B gene from *H. cecropia* was electroporated into channel catfish embryos [28]. In these fish, the expression of the cecropin B gene was regulated by the cytomegalovirus (CMV) promoter in two plasmid constructs. One plasmid was constructed to express the full-length pre-procecropin B, while in the other, the prepro sequence was replaced with the leader sequence from a catfish immunoglobulin VH gene. F_1_ transgenic fish carrying the VH cecropin gene displayed a significantly higher survival rate (40.7%) than non-transgenic fish when both were challenged with *Edwardsiella ictaluri*. The transfer of the pre-procecropin B gene greatly enhanced the survival rate, where 100% of F_1_ transgenic fish survived following a natural epizootic of *Flavobacterium columnare* compared with a 27.3% survival rate in non-transgenic fish. The F_3_ pre-procecropin B transgenic channel catfish exhibited remarkably higher mean survival time when challenged with *Ichthyophthirius multifiliis* than non-transgenic fish [11].

Other immune-related genes were also transferred into fish genomes to enhance their disease resistance. However, the protection level was variable, depending on the fish species and genes transferred. For example, the transfer of human lactoferrin cDNA into grass carp (*Ctenopharyngodon idella*) enhanced resistance to experimental infection with grass carp hemorrhagic virus, as demonstrated by the delayed appearance of clinical signs [91]. Moreover, lactoferrin-transgenic grass carp showed higher resistance to *Aeromonas hydrophila* infection [96]. On the other hand, lysozyme-transgenic Atlantic salmon displayed higher lytic activity in kidney tissue extracts, probably due to higher lysozyme levels from transgene expression [94]. These transgenic fish were not challenged with bacteria to assess their disease resistance [94]. However, the outcomes of the challenge may not be satisfactory, since lysozyme is more effective against Gram-positive bacteria, while the most economically important fish diseases are caused by Gram-negative bacteria [95]. Collectively, these studies demonstrate the effectiveness of antimicrobial and immune-related transgenesis in improving disease resistance; however, this effectiveness depends on the transgene, host species, pathogen type, and the transgene expression level.

### 4.3. Transgenesis for Meat Quality

One of the approaches to enhance the nutritional value of fish meat is to increase its polyunsaturated fatty acid (PUFA) content. The early studies involved the transfer of the genes encoding Δ6-desaturase-like (D6D) and Δ5-desaturase-like (D5D) enzymes from masu salmon (*Oncorhynchus masou*) into the zebrafish genome, increasing the levels of eicosapentaenoic acid (EPA) and docosahexaenoic acid (DHA) in transgenic fish compared with non-transgenic fish [97,98]. Then, trials were conducted on aquaculture species, including the common carp and channel catfish. Cheng et al. [34] generated desaturase-transgenic common carp by electroporating fertilized eggs with the *d5d* gene under the control of the common carp β-actin promoter. The results indicated a 12.7- and 17.9-fold upregulation of *d6d* and stearoyl-CoA desaturase (*scd*) genes in the muscles of transgenic fish fed a commercial diet, while the two genes were downregulated in the liver and brain of transgenic fish compared with non-transgenic control fish. Feeding desaturase transgenic fish on specially formulated diets upregulated *d6d* and *scd* mRNA expression in the muscles and liver, confirming the need for diets customized for desaturase transgenic fish, providing the essential nutrients and substrates for transgenic fish to reach their potential.

In channel catfish, Huang et al. [67] transferred the masu salmon *d5d* gene driven by the common carp beta-actin promoter into one-cell embryos via electroporation. Then, they spawned the P_1_ fish to produce the F_1_ *d5d* transgenic fish. Comparing transgenic F_1_ channel catfish to non-transgenic full siblings, the former displayed a 33% rise in the relative proportion of n-3 fatty acids, a 15% decrease in n-6 fatty acids, and a 17% decrease in n-9 fatty acids. Moreover, several production traits were improved in the *d5d* transgenic channel catfish, including growth, disease resistance, and protein and moisture content in the muscles, not including the fat percentage, which was reduced [67].

Despite these encouraging results, the application of transgenesis to improve meat quality traits in catfish remains underdeveloped compared with the progress made in transgenesis for growth enhancement. For example, the data on sensory meat quality parameters of transgenic catfish, including flavor, texture, and odor, are limited. Similarly, the post-harvest processing characteristics of transgenic catfish were overlooked. Therefore, future research should address these limitations, considering that consumer acceptance ultimately depends on the sensory quality parameters of transgenic fish meat, and fish-processing plants also expect a minimum of carcass quality parameters.

### 4.4. Switching to Fish Sequences

The early transgenic fish were generated using non-fish genes and promoters. For example, the first transgenic fish (goldfish) was generated by transferring the human growth hormone gene. The first growth hormone transgenic catfish contained a human growth hormone sequence fused with the mouse metallothionein promoter. Then, viral promoters, such as the CMV or RSVLTR, were used [28,51]. However, there was a need to use all-fish genes and promoters in generating transgenic fish, a requirement that would facilitate transgenic fish approval and enhance their acceptance by consumers. Since the early 1990s, researchers have focused on developing endogenous fish genes (“all fish” transgenesis) in preference to using mammalian promoters and exogenous genes, such as human growth hormone. For example, an all-fish chimeric growth hormone construct composed of an AFP promoter from ocean pout and Chinook salmon growth hormone cDNA induced 2–6-fold growth enhancement in transgenic Atlantic salmon compared with control fish [27]. Similarly, Devlin et al. [99] developed an all-salmon growth hormone gene construct that accelerated the growth of transgenic coho salmon by over 11-fold. In tilapia, Maclean and Rahman [100] found that carp beta-actin was more efficient than the rat beta-actin promoter in controlling the transient expression of two similar reporter gene constructs. In common carp, an “all-fish” transgenic construct was redesigned using a common carp β-actin promoter to drive the expression of the grass carp growth hormone gene, resulting in remarkable growth enhancement [101].

In addition to transgenesis with natural genes, molecularly engineered (artificial) genes have also been delivered into fish to test their viability. These unnatural sequences can be optimized to ensure compatibility with the host’s codon usage. Also, they can help circumvent the host regulatory mechanisms, since they are completely different from the endogenous sequences. For example, the constitutively activated growth hormone receptor (*ca-ghr*) gene was designed and transferred into zebrafish—a species often resistant to growth enhancement by growth hormone transgenesis. The *ca-ghr* transgenic fish exhibited growth enhancement rates that were impossible with the traditional growth hormone gene transfer [102].

## 5. Limitations of the Traditional Gene Transfer Approach

Although several stable transgenic fish were generated using traditional gene transfer, this approach has limitations in transgene integration, copy numbers, and transgene expression [95]. After introducing the transgene into one-cell embryos, there is no control over subsequent transgene integration events. The transgene may randomly integrate into the genome, including euchromatic or heterochromatic regions [103]. Heterochromatic regions are expected to reduce or inhibit transgene expression [104]. There is also a low possibility that the transgene integrates into a functional gene, resulting in insertional mutagenesis. In some cases, the transgene may persist for long periods in the cytoplasm without integration, where it is replicated and expressed, but will eventually disappear during cell division events. Moreover, multiple transgene copies may integrate into the genome at a single site as tandem repeats or on different chromosomes. For example, Stuart et al. [54] reported about 100 transgene copies per germ cell in a transgenic zebrafish.

The integration time is also variable. Early transgene integration events are desirable to ensure its presence in all cells. Late integration events will result in mosaicism, in which the transgene is present in some tissues. The traditional gene transfer approach typically results in mosaicism; therefore, generating transgenic fish using this approach requires greater effort to screen and select transgenic fish with the desired integration site, transgene copy numbers, optimal expression level, intended germline transmission rates, and improved performance. On the other hand, gene-editing technologies provide easier approaches to generate transgenic fish and overcome these limitations; this includes the potential to mitigate high levels of mosaicism, as discussed in the next section.

The above-mentioned limitations, such as random transgene integration, high mosaicism, unpredictable copy number and expression levels, and the difficulties in screening and selection of appropriate founder fish, are not equal among all the genetic modification strategies employed in catfish research. Traditional transgenesis, CRISPR-assisted insertion, targeted knock-in, gene knockout, and multiplex editing differ significantly in their mechanism, control of integration sites, reliance on double-strand breaks, risk of mosaicism, regulatory classification, environmental risk, and probably also consumer acceptance. These differences are important from both a scientific and regulatory/commercialization perspective. Table 2 provides a structured comparison of these approaches along all these dimensions, with examples from catfish research where available.

## 6. Gene Editing

One of the early efforts to reduce genomic mosaicism involved the co-injection of the I-SceI meganuclease alongside a reporter construct flanked by I-SceI recognition sites. This strategy facilitated the earlier and more efficient integration of the transgene into the host genome. The resulting enhancement significantly reduced the degree of mosaicism and dramatically increased transgenesis frequencies, achieving up to 45% success compared to only 5% when the I-SceI meganuclease was omitted [110,111,112]. This method also allowed for reliable selection of founder fish based on GFP expression, which reflects the quality of injection and transgene integration [110].

In the last decade, advanced gene editing tools have revolutionized fish biotechnology research, including gene knockout and targeted gene insertion (Table 3). The early gene editing research in fish was based on the zinc finger nuclease (ZFN) and transcription activator-like effector nuclease (TALEN) technologies. These technologies rely on two types of protein domains: the first recognizes and binds to a specific DNA sequence (the DNA-binding domain), while the other cuts the DNA strand (the DNA-cleavage domain). ZFNs and TALENs must be designed in pairs, one for each DNA strand, to induce a double-strand break (DSB) in the target gene. The outcome depends on how the cell repairs the DSB. If the DSB is repaired by nonhomologous end joining (NHEJ), indel mutations will occur, resulting in gene disruption or knockout. Genes could be inserted or corrected if the DSB is repaired by homologous recombination in the presence of a DNA template [113]. ZFNs and TALENs have several disadvantages, including higher cost, lower efficiency, difficulty in design, limited genome targets, and relatively higher off-target effects. Now, employing ZFNs and TALENs in fish biotechnology research is mostly replaced by the CRISPR-Cas system (clustered regularly interspaced short palindromic repeats-CRISPR-associated protein), which overcomes the limitations of ZFNs and TALENs.

The CRISPR/Cas9 system is a powerful gene-editing tool that can be designed to target specific genome locations more efficiently than ZFNs and TALENs [114,115,116]. The system uses a guide RNA to target the intended sequence in the genome and an endonuclease enzyme (Cas9) to induce the DSB. Cas9 can cut both DNA strands simultaneously, so only one system is sufficient for one target. Currently, the CRISPR/Cas9 system is the dominant gene-editing tool due to its simplicity, high on-target accuracy (guide RNA-dependent), efficiency, lower cytotoxicity, lower cost, and higher germline transfer of mutations [117,118]. Therefore, most fish biotechnology research has switched from ZFNs and TALENs to the CRISPR/Cas9 system. The various applications of genome-editing tools in catfish and other teleosts are listed in Table 4.

The development of genome editing has progressed beyond the initial CRISPR/Cas9 system to incorporate more recently developed nucleases. Specifically, CRISPR/Cas12a and CRISPR/Cas13 represent powerful, updated tools that enable precise editing of DNA and RNA in aquatic species [126,139,140,141,142,143]. Their distinct functionalities and potential for minimal off-target effects make them highly valuable for applications ranging from trait enhancement to improving disease resistance in fish. For instance, Cas12a has been successfully employed for gene editing in goldfish, targeting genes like tyrosinase (*tyr*). A key finding is that Cas12a activity is highly sensitive to temperature, with a brief application of elevated temperature significantly boosting its efficiency to trigger targeted gene disruption [139].

In fish, gene editing has been achieved for several objectives, such as increasing growth, enhancing disease resistance, improving flesh quality, studying functional genomics, and sterilization [38,43,145,154]. Table 5 summarizes gene-editing research in catfishes.

### 6.1. Gene Editing for Growth Enhancement

The myostatin (*mstn*) gene is the main target gene for growth enhancement in fish using gene-editing technologies. MSTN protein belongs to the transforming growth factor beta (TGF-β) superfamily and is a negative regulator of skeletal muscle growth. Spontaneous mutations in the *mstn* gene were linked to larger muscle mass in several species, a condition known as double muscling. The gene editing of *mstn* in fish focused mostly on gene knockout to disrupt its function and enhance muscle growth. The CRISPR/Cas9 system was successfully employed for growth enhancement in several fish species, including common carp [154], channel catfish [38], medaka [121], red seabream (*Pagrus major*) [163], olive flounder (*Paralichthys olivaceus*) [164], blunt snout bream (*Megalobrama amblycephala*) [122], mud loach [40], Nile tilapia [41], and topmouth culter (*Culter alburnus*) [165].

Several fish species possess two copies of the *mstn* gene (*mstna* and *mstnb*). The first *mstn* gene-edited catfish was a yellow catfish (*Tachysurus fulvidraco*) generated by knocking out the *mstna* gene using the ZFN technology [149]. Then, these fish were bred to generate homozygous *mstna* mutants. The rearing experiment revealed a 1.27- to 1.37-fold improvement in growth of gene-edited fish compared with their control siblings at 80 and 210 days post-fertilization (dpf), respectively. Also, gene-edited fish displayed a double-muscling phenotype as indicated by the hyperplasia of skeletal muscles [39]. The other copy of the *mstn* gene (*mstnb*) was mutated in yellow catfish using the TALENs technology [144]; however, the growth rate did not improve following *mstnb* knockout. On the contrary, *mstnb* knockout negatively affected growth performance, resulting in lower body weight, shorter body length, fewer vertebrae, and reduced intervertebral distances [105]. These results contradict those from blunt snout bream [122], where six-month-old *mstna* and *mstnb* mutants had similar growth rates, both of which were significantly higher than those of wild-type control fish. The analysis of body growth indicators of *mstna*- and *mstnb*-deficient fish revealed higher weight/length and thickness/length ratios, larger average muscle fiber size, and enhanced body type, circumference, and body weight relative to wild-type fish [122]. These contradictions may be attributed to species differences in regard to the evolution of gene function after gene duplication.

The first *mstn* gene-edited channel catfish was generated using the CRISPR/Cas9 system by targeting the *mstnb* ortholog, inducing various indel mutations in the first exon of *mstnb* [38]. These gene-edited fish displayed a 29.7% improvement in growth at 40 dpf compared with their wild-type siblings. In addition, histological examination revealed hyperplasia and hypertrophy in the dorsal muscle of *mstnb*-edited fish compared with wild-type fish [38]. Later, a three-year experiment was conducted to investigate the effects of myostatin knockout across various life stages and rearing environments [157]. In addition to the expected downregulation of *mstnb* gene expression in *mstnb*-edited fish, growth rates were generally higher in edited fish at 12, 18, 24, 31, and 36 months post-hatch than in wild-type controls. Furthermore, an experimental challenge with *E. ictaluri* revealed equal or better survival in *mstnb*-edited fish than in wild-type controls [157].

Beyond myostatin, the melanocortin-4 receptor (*mc4r*) gene represents another target for improving growth performance in fish. MC4R is a transmembrane protein from the G-protein-coupled receptor superfamily that plays a vital role in energy homeostasis and regulation of food intake in teleosts [166,167,168]. Polymorphisms in the *mc4r* gene have been correlated with growth and development in medaka and the cyprinid fish, *Spinibarbus hollandi* [169,170]. In animal models, such as the mouse, knockout of the *mc4r* gene resulted in hyperphagia, obesity, and increased growth [171]. In zebrafish, *mc4r*-knockout fish exhibited higher growth rates after 2.5 months than wild-type fish [172].

In channel catfish, Coogan et al. [106] employed the CRISPR/Cas9 system to knock out the *mc4r* gene, achieving a mutation rate and a germline transmission rate of 33% and 71%, respectively. Their results revealed higher growth rates in *mc4r*-knockouts at various life stages reared in tanks or earthen ponds than the wild-type controls. Gene-edited fish possessing two mutated *mc4r* alleles (homozygous) grew 30% faster than heterozygous mutants in earthen ponds. Similar results were obtained for fingerlings reared in a recirculating aquaculture system. Crossing *mc4r*-mutants resulted in offspring with a 40% enhanced growth rate compared with fingerlings from wild-type crosses. Also, *mc4r* × *mc4r* crosses enhanced the growth rate by 54% compared with offspring from crossing *mc4r* mutants with wild-type fish [106].

To further optimize these editing outcomes, the choice of delivery method also plays a role. The CRISPR/Cas9 system can be introduced into one-cell embryos via either microinjecting gRNA and Cas9 protein or electroporating gRNA- and Cas9-expressing plasmid constructs. Theoretically, microinjecting the gRNA-Cas9 protein complex should induce early mutations compared with using plasmid constructs, which require time for transcription and translation. However, electroporation of plasmid constructs is more convenient and less invasive to the embryos [173]. Therefore, Khalil et al. [107] compared the efficiency of the two methods by targeting the *mc4r* gene in channel catfish via microinjecting a gRNA-Cas9 protein complex versus electroporating gRNA- and Cas9-expressing plasmid constructs. The overall mutation rates were higher for microinjection (66.7–90.6%) than for electroporation (30.6%). Ten-month-old *mc4r*-mutants generated by electroporation grew 38% faster than their wild-type siblings. The feed conversion ratio was also improved in ten-month-old knockouts (1.18) compared with wild-type controls (1.57). The *mc4r*-mutants generated by microinjection displayed a 20% increase in growth at 30 dpf compared with wild-type controls. The two experiments were conducted in different years; therefore, comparing the same-age fish was not possible. However, the 20% improvement in growth at 30 dpf could be magnified when the fish reach the market size. In general, larger initial size in fry and fingerlings correlates with enhanced feed conversion efficiency (FCE) and superior subsequent growth rates, indicating that early developmental advantages are predictive of improved growth performance and nutrient utilization at later life stages.

### 6.2. Gene Editing for Disease Resistance

Gene editing research for enhancing disease resistance falls mainly into two categories: gene knockout and targeted gene insertion of one or more immune-related genes. The gene knockout approach was mostly conducted in tissue culture, revealing promising results, such as reducing viral entry into cells [174], modulating immune signaling pathways [175], and discovering the function of immune-related genes [176]. One of the studies that implemented the gene knockout approach to generate disease-resistant fish focused on the channel catfish toll-interleukin 1 receptor domain-containing adapter molecule (*ticam1*) and rhamnose-binding lectin (*rbl*) genes [158]. These genes are differentially expressed in fish with varying levels of disease resistance. For example, the *rbl* gene was significantly upregulated in a columnaris-susceptible channel catfish strain following *F. columnare* challenge compared with a columnaris-resistant strain [177]. Similarly, the *ticam1* gene was remarkably upregulated in channel catfish, a species more susceptible to *E. ictaluri* infection, compared with a downregulation in blue catfish, a species more resistant to *E. ictaluri* infection [178]. These two genes are potential candidates for gene knockout to study their roles during disease progression and potentially generate disease-resistant fish.

The studies that implemented the CRISPR/Cas9 technologies and assessed the changes in disease resistance in fish were based on targeted gene insertion. Therefore, this section will focus on the targeted insertion of immune-related genes with effects measured in fish. In traditional gene transfer approaches for enhancing disease resistance, a transgene is introduced into one-cell embryos via microinjection or electroporation to randomly integrate into the recipient fish genome, which has several disadvantages, as discussed earlier. Gene editing-mediated targeted gene insertion allows for control over the insertion site and the transgene copy numbers [109]. One of the potential target sites for transgene insertion is the non-coding regions of the fish genome, avoiding insertional mutagenesis and selecting the best sites for transgene expression. The first CRISPR/Cas9-mediated gene insertion in channel catfish was conducted by Simora et al. [108], where the researchers inserted a cathelicidin gene (*cath*) from the American alligator (*Alligator mississippiensis*) into a non-coding region on chromosome 1 of channel catfish. Cathelicidins are a group of antimicrobial peptides with strong activity against Gram-negative bacteria and multidrug-resistant pathogens, including *Klebsiella pneumoniae* and *Acinetobacter baumannii* [179]. In the study by Simora et al. [108], integration rates were generally higher in dead fry than in alive fry, indicating possible pleiotropic or off-target effects. Also, high expression of *cath* was detected in several tissues of P_1_ fish, including muscles, gills, stomach, heart, kidney, fins, and eyes.

Until recently, gene editing research in catfish focused on single genes or genes with similar functions, i.e., genes for growth enhancement or disease resistance, using either gene knockout or targeted gene insertion. Combining the two approaches to insert and knock out genes simultaneously in a single fish shortens the time for genetic experiments and maximizes the benefits of gene editing. A recent study inserted the *cath* gene from the Chinese alligator (*Alligator sinensis*) (*As-cath*) into the blue catfish luteinizing hormone (*lh*) locus via two CRISPR/Cas9 systems to enhance disease resistance [44]. The on-target knock-in (KI) rates of the two CRISPR/Cas9-assisted systems were 10.26 and 16.04% for the linear double-stranded DNA (dsDNA) and the double-cut plasmid, respectively. In addition, the *As-cath* gene was expressed in nine tissues of P_1_ fish, including the kidney, skin, and muscles. Most importantly, the *As-cath* transgenic blue catfish exhibited a remarkable survival rate (80%) after *Flavobacterium covae* infection compared with wild-type control fish (30%), with no pleiotropic effects on growth rate or external morphology [44]. The impact of KI of *As-cath* in the *lh* locus was not reported for blue catfish. However, it is expected to block LH function via insertional mutagenesis, as shown in a similar study on channel catfish [109].

Wang et al. [43] used the CRISPR/Cas9 system to insert the *As-cath* gene into the *lh* locus of channel catfish to generate sterile disease-resistant fish. The researchers used two delivery systems: one with dsDNA and another with single-stranded oligodeoxynucleotides (ssODNs). dsDNA was more efficient at inducing on-target KI of *As-cath* (10.8%) than ssODNs, which induced only one KI case. They observed several outcomes in treated fish, including on-target KI of *As-cath* with *lh* knockout (LH^−^-As-cath^+^), off-target KI of *As-cath* without *lh* knockout (LH^+^-As-cath^+^), and *lh* knockout without *As-cath* KI (LH^−^-As-cath), with LH^−^-As-Cath^+^ being the most desired outcome. The P_1_ founders were mosaic, as confirmed by transgene expression across different tissues and germline transmission to the offspring. Disease challenges with *E. ictaluri* and *F. covae* revealed significantly higher survival in *As-cath*-transgenic fish than in wild-type controls. Fertility was also restored after hormone therapy, indicating a breakthrough in generating disease-resistant fish in which fertility could be restored only by man, thereby preventing potential ecological concerns associated with these transgenic fish [43].

Gene editing for disease resistance and growth enhancement could also be integrated to generate disease-resistant, fast-growing fish. A good example is combining antimicrobial peptide gene (*cath* and *cec*) transgenesis with *mstn* or *mc4r* knockout. Wang et al. [180] utilized the CRISPR/Cas9 technology to insert *cath* and *cec* into three loci in the channel catfish genome, including *mstnb*, *mc4r*, and *lh*. The transfer of *cec* enhanced the resistance to *E. ictaluri* twofold, with further improvement from the simultaneous KI of *cath*. Knock-in of *cec* combined with *mstn* knockout (KO) improved both survival by threefold following the challenge with *E. ictaluri* and growth by 50% in four-month-old fish. Insertion of *cath* at the *lh* locus resulted in a fourfold increase in resistance to *F. covae* and a 10% reduction in growth at two years of age. Simultaneous KI of *cath* with *mc4r* KO reversed the negative effects on growth, achieving 80% growth enhancement and 2.5-fold improvement in *F. covae* resistance in three-month-old fish. Combining *cath* and *cec* KI with *mstnb* and *mc4r* KO induced a four-fold increase in resistance to *E. ictaluri* coupled with a 50% growth enhancement in three-month-old fish [180]. Future studies should investigate the long-term effects, evaluate other pleiotropic effects, and conduct selective breeding to maximize the benefits of combined gene insertion and knockout.

### 6.3. Gene Editing for Meat Quality

Most previous gene-editing studies for growth enhancement did not assess the direct or pleiotropic effects on meat quality or carcass composition of fish. However, it would be interesting to investigate these impacts as gene insertion or knockout has been shown to affect more than a single trait. In fish, gene editing to enhance meat quality focused on two main approaches: (1) promoting consumer acceptance of certain fish species by preventing intramuscular bone (IB) development and (2) increasing the nutritional value of fish meat by elevating PUFA content.

IBs have several negative impacts, including choking hazards for consumers, difficulty in fish processing, and reduced commercial value of fish products. Therefore, many researchers attempted to develop IB-free fish using various genetic techniques, such as selective breeding, interspecific hybridization, and gynogenesis (reviewed in Nie et al. [181]). However, none of these approaches completely removed IBs except in one gynogenetic grass carp fish [182]. Recently, researchers employed the CRISPR/Cas9 technology to knock out the genes responsible for IB formation and produce IB-free fish, including zebrafish [183], blunt snout bream [184], Gibel carp [185], and crucian carp [123]. Catfish do not contain IBs; therefore, we will focus on enhancing PUFA content in their flesh.

Marine fish species, such as Atlantic salmon, can synthesize PUFA, while most freshwater fish species have low PUFA content. Several studies employed CRISPR/Cas9 to unveil the genes involved in PUFA synthesis. For example, researchers knocked out the very-long-chain fatty acyl elongases (Elovls) in Atlantic salmon using the CRISPR/Cas9 system [186]. Their results demonstrated lower PUFA levels in the liver, brain, and white muscle of *elovl2* KO fish, revealing the vital role of *elovl2* in PUFA synthesis. In addition, the roles of other genes, such as *d5d* and *d6d*, in PUFA synthesis were also discovered by gene knockout [187]. These genes were transferred into the channel catfish genome to increase the PUFA content.

Previously, the masu salmon *d5d* gene, driven by the common carp beta-actin promoter, was transferred to channel catfish using traditional gene transfer methods [67]. With the advent of CRISPR/Cas9, researchers inserted several candidate genes, individually or combined, into specific targets in the channel catfish genome to elevate PUFA content and achieve other benefits of gene editing. An early CRISPR/Cas9-based trial involved inserting the masu salmon *elovl2* gene driven by the common carp beta-actin promoter into exon 2 of the channel catfish *lh* gene [45]. The authors reported a 37.5% integration rate with transgene expression detected in 12 tissues of P_1_ fish, including the liver (13.4-fold) and muscles (9.2-fold). Interestingly, the n-3 and n-6 PUFAs were notably elevated in transgenic fish compared with non-transgenic controls.

The *elovl2* gene was also integrated into a non-coding region on channel catfish chromosome 1 using the CRISPR/Cas9 system compared with random integration [159]. The CRISPR/Cas9-mediated integration rate (19%) was lower than random integration (27.3%), whereas mosaicism in P_1_ fish was reduced in fish generated by CRISPR/Cas9 compared with random integration, suggesting a higher germline transfer rate of transgenes. Importantly, *elovl2* expression was significantly higher in most tissues of CRISPR/Cas9 P_1_ fish, accompanied by a 20.7% improvement in DHA content [159]. Another study investigated the channel catfish *mc4r* gene as a target locus for inserting the masu salmon *elovl2* gene, elevating the PUFA content and improving the growth rate (41.8%) in six-month-old *elovl2*-transgenic *mc4r* KO fish [127].

In addition to the masu salmon *elovl2* gene, other candidate genes, such as the *Δ6 fad* from rabbitfish (*Siganus canaliculatus*), were successfully inserted into the long repeated sequences of the channel catfish genome to enhance KI efficiency and integrate multiple transgene copies [160]. CRISPR/Cas9 was also utilized to insert codon-optimized *fat-1* and *fat-2* genes from *Caenorhabditis elegans* into long repeated sequences of channel catfish [46]. The *fat-1* gene encodes an n-3 fatty acid desaturase, whereas the *fat-2* gene encodes the delta12 fatty acid desaturase. The integration rate was 9.1% for *fat-1*, a three-fold higher rate than *fat-2*. Transgenic fish carrying the *fat-1* and *fat-2* genes exhibited higher expression levels of endogenous genes *fads6*, *elovl2*, and *elovl5*. Interestingly, the content of DHA, total n-3, and n-3/n-6 ratios increased by 37%, 19.8%, and 25.2%, respectively [46].

## 7. Pleiotropic Effects of Transgenesis and Gene Editing

Inserting a transgene into fish genomes may result in pleiotropic effects. These effects occur when a transgene impacts one or more traits positively or negatively [95]. These pleiotropic effects arise from altering the functions of one or more endogenous genes [188]. Introduction of transgenic constructs into channel catfish embryos has been shown to reduce survival and hatchability [189]. In addition, transgene insertion can induce global gene expression changes, as demonstrated by Han et al. [190], who studied the gene expression changes in the spleen, liver, and kidney of cecropin P_1_ transgenic rainbow trout, suggesting that cecropin transgenesis may not only eliminate pathogens directly but also exert immunomodulatory effects to boost the fish’s immune responses. Another explanation, although less likely, is that the transgene may be randomly inserted into a functional gene, resulting in mutagenesis. If this is the case, the phenotype of the transgenic host will be a combination of the gene insertion and knockout effects. Therefore, a complete assessment of the pleiotropic effects of transgenes is necessary for each transgene and fish host.

In addition to growth enhancement, several non-specific immune parameters were improved in the growth hormone transgenic common carp, including lysozyme activity, serum bactericidal activity, spleen weight, leukocrit value, and phagocytic percent of macrophages in the head kidney [191], resulting in higher resistance to experimental infestation by *I. multifiliis* [192]. Conversely, growth hormone transgenic coho salmon exhibited lower resistance to experimental *Vibrio anguillarum* infection [193] and were more susceptible to predation than non-transgenic fish [194]. Similarly, growth hormone transgenic channel catfish fingerlings were more vulnerable to predation by largemouth bass (*Micropterus salmoides*) and green sunfish (*Lepomis cyanellus*) than non-transgenic control fish [195].

At the cellular level, RSVLTR-rtGH transgenic channel catfish had more mitochondria, glycogen globules, and muscular fibers, and fewer fat globules than wild-type controls [196]. The size of the muscle fibers was not altered. In addition, transgenic catfish meat had a slightly superior flavor and texture than non-transgenic controls, which could be attributed to alterations in amino acid levels and ratios, muscle fat, and ultrastructure. Similarly, the third-generation growth hormone-transgenic Mutiara catfish exhibited better feed efficiency, elevated protein content, and higher content of essential amino acids, especially methionine and lysine, than non-transgenic control fish [88]. Moreover, the fourth-generation growth hormone-transgenic Mutiara catfish performed similarly to the third generation, except for the higher expression of insulin-like growth factor 1 (*igf1*) [89].

Survival was also altered by growth hormone transgenesis. At −0.5 °C, survival was enhanced (100%) in growth hormone transgenic channel catfish fry (opAFP-ccGH) compared with 0% survival in their full-sibling control fish, CB hybrid catfish, and glutamic acid decarboxylase 65 (*gad65*) transgenic catfish [197]. In addition, survival at 22 to 27 °C and 7.5 ppt salinity was significantly higher (66.67%) in channel catfish fry transgenic for the growth hormone constructs opAFP-ccGH and rtMT-ccGH than in non-transgenic full-sibling control fish (10.07% for opAFP-ccGH control and 12.82% for rtMT-ccGH control) [198].

Pleiotropic effects were not limited to growth hormone transgenes. The transfer of the *d5d* gene from masu salmon to channel catfish improved PUFA content and induced various pleiotropic effects on growth, disease resistance, and tolerance to hypoxia [67]. This transgene favorably influenced body weight, where three transgenic families combined had remarkably larger body weights than non-transgenic fish. Also, the effects on tolerance to hypoxia were variable; resistance increased in some transgenic families and decreased in others, probably due to family effects, transgene integration sites, or copy numbers. For disease resistance, *d5d*-transgenic fish survival following natural *F. columnare* infection was significantly better than that of non-transgenic full-sibling controls, which experienced 52.9% mortality [67].

Similar to transgenesis, gene knockout can also result in pleiotropic effects. In gene knockout, indels are usually introduced in the coding sequence of a gene, leading to a frameshift mutation or a premature stop codon [118]. The resulting protein will either be truncated or have a different sequence from the wild-type protein, and in both cases will not function properly, leaving a gap in the corresponding pathway(s) and affecting the regulatory mechanisms. Some species mitigate the impact of knockout by activating alternative pathways or adjusting the production levels of other proteins via feedback mechanisms. For example, *mstn* knockout in medaka significantly increased body weight and altered the expression of genes related to myogenesis and immune responses [199,200]. Therefore, gene knockout will likely have an impact unless the host genome contains duplicate copies of the gene, and not all of them were knocked out, as in salmonids [201].

The pleiotropic effects of gene knockout may be positive or negative. For *mstn* knockout, most studies reported favorable pleiotropic effects, except in medaka. TALEN-mediated *mstn* knockout in medaka enhanced growth rate by 25% at the post-juvenile stage at the expense of immune response. Following an immersion challenge with red-spotted grouper nervous necrosis virus, *mstn*-KO medaka exhibited higher virus copy numbers than their wild-type controls [199]. Moreover, *mstnb* knockout in yellow catfish was associated with reduced body weight, shorter body length, reduction in vertebral number, and reduction in intervertebral distance, suggesting that this myostatin paralog is involved in skeletal development apart from muscle growth, and that the knockout of one paralog may not be fully compensated by the remaining copy. On the other hand, *mstnb*-KO channel catfish exhibited higher body weights with equal or better resistance to *E. ictaluri* than wild-type controls [157]. Knockout of *ticam1* and *rbl* genes in channel catfish resulted in congenital anomalies in some four-month-old fingerlings, including the absence of eye development, absence of barbels, and head and spinal deformities [158]. These anomalies were not observed in control non-injected fish or control fish injected with buffer (water and phenol red), suggesting they could be due to gene knockout or off-target effects. Also, channel catfish embryo survival decreased with increasing the CRISPR/Cas9 dosage injected at the one-cell stage [158].

Most pleiotropic effects of gene editing in fish were evaluated primarily by examining host performance or measuring the expression of individual genes, which may not provide a complete picture of how unintended effects arise. Genome editing can result in allele dropout, mosaicism, and tissue-specific genotype discrepancies, which can lead to phenotypic outcomes that differ across tissues or developmental stages. In addition, genome editing can result in large deletions, inversions, chromosomal rearrangements, unintended donor integration, and concatemer formation [202,203,204].

Off-target effects may be considered a possible cause of pleiotropy; therefore, genome sequencing could demystify the nature and locations of these off-targets. This includes targeted amplicon sequencing to confirm on-target modification, followed by whole-genome and long-read sequencing to detect structural variants, with a clear distinction between P_1_ founder screening and F_1_/F_2_ germline validation. Another approach is to study gene expression changes of gene editing in specific tissues of interest using RNA-Seq. Both approaches could reveal the developmental trade-offs and compensatory effects—mechanisms by which disruption of one gene stimulates compensatory response in related genes or pathways—which represent an underexplored area of genome-editing safety in fish.

## 8. Potential Risks and Ecological Concerns

Reproductive performance, foraging capacity, and predator evasion are important aspects in assessing the viability of transgenic fish and should be assessed before commercialization. According to the majority of existing research, transgenic fish are less fit than non-transgenic fish and are unlikely to have a significant impact on the ecosystem [15,194,195,205].

Transferring salmonid growth hormone genes to channel catfish boosted their growth rate by 33% relative to non-transgenic fish in ponds with additional nutrition. However, without supplementary feeding, the growth rate did not differ between transgenic and non-transgenic channel catfish in ponds, implying similar foraging capacity and transgenic catfish’s failure to exhibit the potential to grow due to insufficient feed [195]. When raised outdoors with limited feed, the transgenic channel catfish showed somewhat lower survival rates than the non-transgenic fish but grew at a comparable speed as the controls.

Dunham et al. [195] conducted the first risk assessment studies using transgenic fish in a natural aquaculture environment. They evaluated the predator evasion skills and growth abilities of transgenic catfish in ponds and discovered that non-transgenic channel catfish fry outperformed the transgenic genotype. Furthermore, there was barely any difference in growth rate between transgenic and non-transgenic channel catfish in ponds with no additional nutrition. The results suggested that transgenic channel catfish could be utilized in aquaculture businesses without harming the natural ecosystem. While transgenic channel catfish may be accidentally released into nature, any ecological impact is improbable because their greater vulnerability to predators would most likely reduce or remove them.

In channel catfish, growth hormone transgenesis did not influence reproduction, as transgenic fish could reproduce and reach sexual maturation at roughly the same pace as non-transgenic fish [65]. The spawning success of transgenic and non-transgenic channel catfish seemed to be equal. Mating occurred randomly, and the spawning performance was similar when the two genotypes were communally stocked in a pond [206].

These ecological and reproductive findings have important regulatory implications. The approval of gene-edited fish appears to be much easier than that of transgenic fish. It took approximately 25 years to approve the first transgenic animal, the growth hormone transgenic Atlantic salmon, for human consumption. On the other hand, four gene-edited fish were granted clearance for commercial production and human consumption in Japan (*mstn*-edited red seabream, leptin receptor (*lepr*)-edited tiger pufferfish, and *lepr*-edited olive flounder), Brazil (*mstn*-edited Nile tilapia), and Argentina (*mstn*-edited Nile tilapia) a few years after they were generated [207]. Japan, Brazil, and Argentina considered these gene-edited fish non-genetically modified organisms (non-GMO); therefore, they bypassed the long process of transgenic regulations [208]. In Europe, the case is different. The European Court of Justice has ruled that gene-edited products must be subject to GMO regulations [209]. Regardless, a complete investigation of the ecological impact of gene-edited fish is necessary, including the pleiotropic effects and long-term environmental risk assessment.

## 9. Transgenic Sterilization

The key ecological concerns about using transgenic fish are the loss of genetic diversity, biodiversity, and species abundance. The most efficient approach to avoiding reproductive communication with wild populations is establishing full infertility in the transgenic breeding population. Triploidy was one of the first methods used to sterilize transgenic fish and is now widely used in AquAdvantage salmon. However, this approach is not suitable for catfish because of several limitations. Triploidy does not always result in complete sterility, and its effect on performance varies with the rearing environment, with several studies reporting reduced overall performance [196,210,211,212]. After six months of rearing in earthen ponds, triploid and diploid channel catfish exhibited similar harvest weight and dress-out percentage; however, triploid fish demonstrated lower survival, reduced yield, and higher feed conversion ratio [210]. Also, environmental risk is not eliminated by triploidy because of the need for fertile broodstock. Moreover, applying triploidy in catfish on a commercial scale is labor-intensive.

Given the limitations of triploidy, transgenic sterilization approaches could provide a means for more reliable sterilization, which would almost eliminate ecological risks, one of the most critical factors in approving transgenic fish for commercial aquaculture. These approaches aim to generate sterile transgenic fish whose fertility can be restored under controlled conditions using chemicals during embryonic development, ensuring that these fish will not reproduce without human intervention.

Several approaches for generating transgenic sterile fish were attempted with variable success rates depending on the technology and the fish species (Table 6). One prominent approach involves transgenic RNA interference (RNAi) coupled with repressible promoters to knock down primordial germ cell (PGC) genes. For example, Li et al. [213] investigated salt-sensitive Tet-Off-like systems in channel catfish, using zebrafish adenylosuccinate synthase 2 (*adss2*) and racemase (*rm*) promoters to drive double-stranded short hairpin RNA (ds-shRNA) constructs targeting *nanos* and *dnd*. Sodium chloride (4 ppt) was used as a repressor to regulate sterility. Their results showed spawning rates of 93% in treated (fertile) P_1_ fish versus 59% in untreated (sterile) counterparts, indicating successful sterilization and repression [213]. However, the study highlighted a critical limitation: the promoters’ sensitivity to naturally occurring salinity levels could allow escaped transgenic catfish to reproduce in estuarine environments, undermining containment efforts. Similarly, Li et al. [214] explored copper-sensitive promoters, such as yeast *ctr3* and a reduced version (*mctr*), paired with RNAi constructs targeting *nanos* and *dnd*. Copper sulfate repressed the knockdown, with untreated transgenic F_1_ fish exhibiting reduced gonad development (93.4% smaller in females, 92.3% in males) compared to non-transgenic controls [214]. Despite these successes, pleiotropic effects, including reduced body weight (25.8% in females, 41.9% in males), suggest unintended consequences that could impact commercial viability. The overexpression of full-length *nanos* complementary DNA (cDNA) has also been tested to disrupt PGC development. Li et al. [213] reported that this strategy, driven by salt-sensitive promoters, reduced *nanos* expression in untreated embryos, leading to sterility. However, the approach showed variable success compared to ds-shRNA methods, suggesting that RNAi-based knockdown may be more efficient [213].

Another transgenic sterilization approach involved transferring the glutamic acid decarboxylase 65 (*gad65*) gene from goldfish to one-cell channel catfish embryos to disrupt gonadotropin-releasing hormone (GnRH) neuron migration and generate reversible sterile fish [35]. At five years of age, F_1_ transgenic fish exhibited a lower sexual maturity score than control fish. The results of natural spawning experiments indicated a generally lower spawning success in transgenic fish than in wild-type controls of the same age. Interestingly, luteinizing hormone-releasing hormone analog therapy restored fertility in two-thirds of the putative infertile transgenic fish [35]. The uniform sterility of all transgenic individuals was not attained, even though this procedure produced reversible sterility in channel catfish. This suggests that the methodology does not yet deliver complete or consistent sterility across individuals. To reach the degree of reproductive containment required for large-scale aquaculture, ongoing optimization, the creation of substitute technologies, or integration with selective breeding programs would be necessary.

A third transgenic sterilization approach tested in channel catfish involved the overexpression of the pro-apoptotic gene *bax* in germ cells through embryo electroporation of three transgenic constructs: *nanos-bax-nanos*, *nanos-bax-dnd*, and *dazl-bax-vasa* [36]. Ye et al. [36] declared that the sterilization of P_1_ females through *bax* overexpression exhibited low efficacy, potentially attributable to a higher tolerance to apoptosis in female germ cells. Additionally, the anticipated repression of transgene expression via doxycycline treatment during embryogenesis was not achieved, which may be due to the continuous expression of the *bax* gene throughout gametogenesis. This technology demonstrates potential for the generation of sterile male fish in the P_1_ generation and likely sterile male and female fish with complete primordial germ cell (PGC) ablation in the F_1_ generation; however, the repression of this process with doxycycline treatment is considered impractical for producing food fish [36].

While the previous transgenic sterilization approaches provided variable levels of sterility, direct disruption of reproductive hormone genes offers permanent sterilization that does not rely on inducible promoters or the use of chemicals. These genes include *lh*, *gnrh*, follicle-stimulating hormone (*fsh*), or germ cell development factors (e.g., *dnd1*). GnRH regulates the reproductive axis by stimulating gonadotropin release, while LH drives gametogenesis. Knockout of these genes can halt reproductive development, producing sterile fish [148]. Therefore, Qin et al. [148] used ZFNs to target the *lh* gene in channel catfish. Electroporation of ZFN constructs into embryos resulted in sterility, with treated fish showing underdeveloped gonads. While effective, this method lacks repressibility, limiting its flexibility for broodstock management compared to RNAi-based systems [148]. Nonetheless, it demonstrates the potential of precise gene editing for permanent sterilization.

In addition, Qin et al. [145] applied TALENs to the *cfgnrh* gene, delivering plasmids via double electroporation. Mutagenesis occurred in 52.9% of P_1_ fish, with sequence alterations (deletions, substitutions, insertions) reducing spawning rates to 20–50% and hatch rates to 2–66% compared to controls [145]. TALENs’ single-nucleotide specificity and reduced off-target effects improved efficiency over ZFNs, though delivery challenges persisted due to the large TALEN constructs. TALENs’ advantages include ease of design and higher specificity, but their application in catfish sterilization remains underexplored. The technology’s complexity and cost relative to CRISPR/Cas9 have shifted research focus, limiting comprehensive data on its full potential in this context [216,217,218].

Moreover, Qin et al. [156] targeted the *fsh* gene by employing the double electroporation technique to transfer TALEN-expressing plasmids into one-cell channel catfish embryos. Knockout of the *fsh* gene in P_1_ fish reduced the natural spawning rates to 33.3–40.0% without hormone therapy. However, the spawning rate of P_1_ mutants was improved to 80.0% in females and 88.9% in males following the injection of human chorionic gonadotropin hormone, indicating the feasibility of restoring reproductive capacity by hormone therapy [156].

While early studies in catfish targeted individual genes, there has been growing interest in editing multiple genes recently. Multiple gene editing could be used to enhance more than one economic trait while achieving sterility simultaneously. For example, Wang et al. [43] combined sterilization with disease resistance by integrating an alligator *cath* gene into the *lh* locus of channel catfish using CRISPR/Cas9. The resulting gene-edited catfish were sterile unless treated with reproductive hormones, thereby ensuring containment while enhancing resistance to bacterial pathogens such as *F. covae* and *E. ictaluri* [43]. This dual-trait approach exemplifies the multifunctionality of CRISPR in aquaculture [109]. The integration of sterilization, disease resistance, and growth enhancement in catfish via CRISPR holds immense potential for sustainable aquaculture. Therefore, future research should focus on refining multi-gene editing strategies, such as multiplexed CRISPR systems, to target *lh*, *mstn*, *mc4r* genes, and *As-cath* & *cec* transgenes simultaneously.

## 10. Recommendations and Future Prospects

### 10.1. Transcriptomics in Phenotypic Assessment of Transgenic and Gene-Edited Fish

Despite significant progress in enhancing catfish growth through transgenesis and gene editing, a major limitation in evaluating the effects of gene insertion or knockout exists. Most current literature relies on studying the presence/absence of genetic change, with a few studies reporting the expression of individual genes following gene insertion or knockout. A fundamental area that remains unexplored is the effect of transgene insertion or gene knockout on global gene expression. Transgene insertion is not a transcriptionally neutral event. In contrast, it can induce changes in the transcriptome, not only in the organ in which it is expressed but also globally. For example, the insertion of cecropin has been shown to induce global transcriptomic changes in rainbow trout, particularly in genes related to innate and adaptive immune responses [190]. Similarly, transcriptional changes are also expected when a gene is knocked out or edited, especially in the signaling pathways in which it is involved. Therefore, studying the global gene expression of transgenic and gene-edited catfish using RNA sequencing would help understand how the transgene effect is mediated, how gene knockout influences signaling pathways, and what other candidate genes play a significant role in the phenotype of interest. Studying the transcriptome could also reveal the alternative pathways that may be modulated in these fish. It could also be used to identify candidate genes for disease resistance, considering that diseases cause more economic damage than any other factor affecting production. However, bulk RNA-seq alone has limitations: mRNA abundance does not reliably predict protein abundance, bulk tissue samples hide cell-type-specific responses, and transcriptional changes do not by themselves demonstrate causation. Future studies should therefore complement bulk transcriptomics with single-cell and spatial transcriptomics to resolve tissue- and cell-type-specific responses, proteogenomics to bridge the gap between transcript and protein abundance, and multi-omics integration with causal network inference to distinguish regulatory factors from downstream effects of transgene insertion or gene knockout in catfish.

### 10.2. Addressing Compensatory Mechanisms

Research evidence has suggested that multiple genes control traits such as growth, disease resistance, meat quality, and reproduction. For example, vertebrate growth is mediated by several growth factors, including MSTN, GHR, GH, IGF-1, etc. These multiple genes function in several signaling pathways, with the possibility of redundant or compensatory mechanisms, as reported in zebrafish reproduction [219]. Disruption of any of these genes could upregulate others. Therefore, we recommend that future research investigate the activation of compensatory pathways. This could be achieved through whole transcriptome profiling to identify upregulation of related genes. This could be further strengthened through proteomics, in which proteins are the workhorses inside the cell, considering that transcriptional changes are not always reflected at the protein level. If compensatory mechanisms are detected for a trait of interest, then multiplexed gene editing targeting the primary gene and its key compensators should be evaluated.

### 10.3. Gene-Environment and Gene-Nutrition Interactions

Genotype × environment (G × E) interactions are well established in aquaculture, where the best-performing genotype for one environment may not be the best for another. Transgenic and gene-edited fish produced in a certain environment should be evaluated under different conditions. This includes the rearing system, water quality, pathogen pressure, and stocking density. Nutrition is equally important, as the potential of gene-edited fish cannot be reached with sub-optimal diets. Dietary variables including protein levels and sources, lipids, and energy should also be evaluated. For example, a growth-enhanced *mstn*-knockout catfish strain may have different protein requirements, in terms of quality and quantity, than wild-type fish and may not reach its growth potential with commercial diets. Similarly, a gene-edited fish for meat quality improvement may also require customized aquafeeds. Future studies should consider environmental variables and dietary needs to evaluate and improve the performance of these fish. A proposed thorough assessment can be fulfilled using factorial experimental designs that simultaneously evaluate many factors and their interactions in order to find the environmental and nutritional circumstances under which these fish lines reach their full productive potential.

### 10.4. Advanced Gene Editing Tools

The majority of gene editing research on catfish has been conducted using the CRISPR/Cas9 system. Although it outperformed TALENs and ZFNs, the possibility of off-target effects could not be excluded. Therefore, utilizing more advanced gene-editing tools in catfish represents a logical next step. These tools include base editors and prime editors. Base editors are used to replace a single nucleotide with another without inducing double-strand breaks [220]. In contrast, prime editors introduce small insertions, deletions, or substitutions in the genome, also without double-strand breaks [221]. The current use of prime editors in fish is limited to model organisms such as zebrafish and has not been investigated in commercial fish species [221]. These advanced tools minimize the risk of off-targets, increase the specificity of targeted gene editing, reduce the need for whole-genome sequencing to identify off-target effects, and deserve consideration in future catfish gene-editing studies.

### 10.5. Integration of Genomic Selection and Artificial Intelligence

Genetic modification through transgenesis and gene editing should not be viewed as a replacement for conventional selection or genomic selection. In contrast, it should be considered as a complementary toolkit to accelerate genetic improvement of economically important traits, especially when natural genetic variation, the prerequisite for selection, is limited. Genomic selection predicts the estimated breeding values for individual candidates, enabling more accurate selection decisions for polygenic traits—traits that are controlled by multiple genes or loci with small individual effects such as growth, disease resistance, and fillet quality—although some of these traits also include identified large-effect loci, such as *mstn* and *mc4r* for growth [38,39,41,42,106,107]. Therefore, genomic selection remains the primary and most broadly applicable tool for selection of superior individuals across these polygenic traits. On the other hand, gene editing is preferred for targeting specific, well-characterized genes with large effects. The most efficient genetic improvement program of the future is the one that combines both approaches: genomic selection for continuous incremental improvement of polygenic traits and gene editing for precise modifications of large-effect genes. Genomic selection and gene editing are not mutually exclusive and can operate on different levels of genetic improvement programs. For example, a catfish line carrying a myostatin knockout or a *cath* knock-in could serve as a founder population for genomic selection to improve polygenic traits, such as fillet quality. This integrated strategy maximizes genetic gain across both simple and complex traits.

Selection could also be implemented within the transgenic or gene-edited populations to improve the edited trait of interest. The outcomes of transgenesis or gene editing are not identical among individuals of the parental generation due to mosaicism, different indel compositions, and genetic background. This was observed in *mstn*- and *mc4r*-edited channel catfish, in which body weights exhibited high variability [107,157], similar to the observation in growth hormone-transgenic catfish [82]. Therefore, future studies should consider applying selection to transgenic or gene-edited populations to augment performance gain and shorten the time to improve the traits of interest.

Artificial intelligence (AI) and machine learning (ML) have the potential to revolutionize the design of gRNA and the prediction of off-targets, thereby improving the efficiency and accuracy of genome editing in aquaculture. The currently available deep learning models have been shown to outperform traditional algorithms in gRNA design and off-target prediction [222], but these models were developed and tested for mammalian systems, such as humans and mice; therefore, they may not perform to the same standards on catfish genomes, especially when accounting for chromatin accessibility and complex thermodynamic interactions.

**Figure 2:**
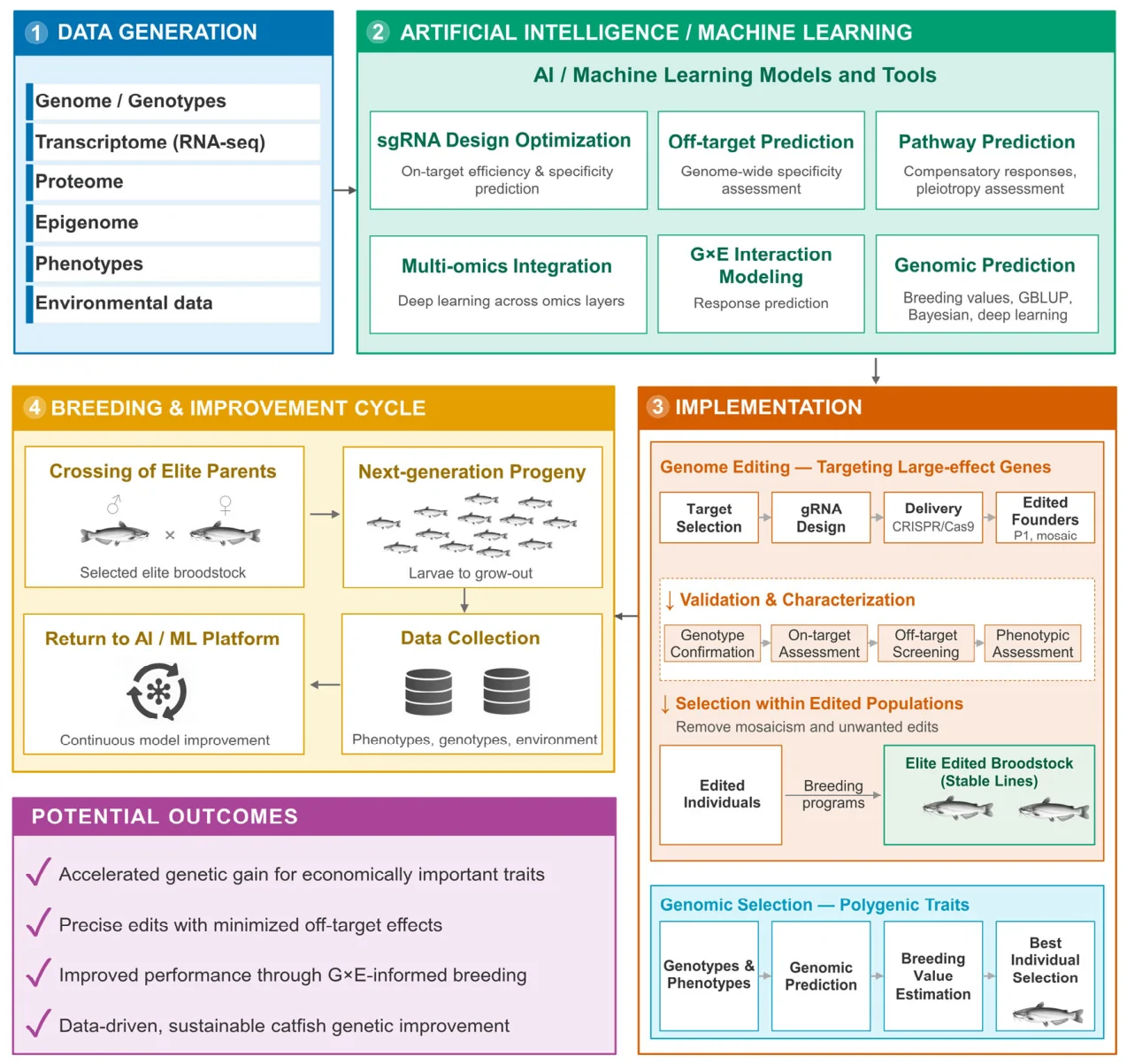
Integrated artificial intelligence/machine learning (AI/ML)-assisted genetic improvement framework for catfish. This framework includes four major components: (1) Data generation at various levels, beginning with genotypic and genomic data to environmental data. (2) The AI/ML component for data processing. (3) The implementation stage, which has two parallel tracks: genome editing of large-effect genes and genomic selection for quantitative traits controlled by polygenes. (4) The breeding and improvement cycle, in which elite edited strains are developed. G: genotype; E: environment; ♂: male; ♀: female.

Finally, AI-assisted multi-omics research could integrate data from genome-wide association studies (GWASs), tissue-specific expression quantitative trait loci (eQTL), protein interactions, and predicted compensatory pathway responses to identify target genes that are most likely to generate the desired phenotype with little or no off-target effects [223,224]. In genomic selection, machine learning methods that can capture non-additive genetic effects and G × E interactions may further enhance the precision of breeding value estimation in catfish populations with complex genotype-environment dynamics [225]. Therefore, future studies should prioritize developing and benchmarking ML models for catfish gRNA optimization and off-target prediction, with ML models trained on catfish datasets to support catfish genetic enhancement programs (Figure 2).

### 10.6. Public Education

One of the challenges preventing the impact of genetic biotechnology is public education [226]. With increasing urbanization, it is difficult for the public to understand where their food originates [227]. In addition, highly educated people have difficulty comprehending genetic and molecular biotechnology [228]. Social science research demonstrates that consumer attitudes toward transgenic and gene-edited organisms are influenced by several factors, including trust, transparency, product labelling, perceived naturalness, animal welfare, environmental concerns, corporate ownership, and cultural values [204,229,230,231,232,233]. Thus, increased and transparent public education about the benefits and risks of genetic biotechnology is urgently needed to gain public acceptance of the remarkable gains in the genetic enhancement of catfish through transgenesis and gene editing. To further foster public acceptance of transgenic and gene-edited catfish and other aquatic organisms, greater emphasis should also be placed on addressing traits important to consumers, such as nutritive value, texture, and flavor.

## 11. Conclusions

This review provides critical insights into catfish transgenesis and gene-editing research, from the generation of the first transgenic catfish species to the identification of the current research gaps and suggestions for future research. The current literature shows that significant progress has been made in transgenesis and gene editing in catfish; however, most of this progress has been focused on North American catfish species, including the channel catfish. Future research should invest in the many other commercially important catfish species that millions of people worldwide depend on for food and livelihoods.

In particular, gene editing has proven to be a great tool for sterilizing catfish. This sterility has been overcome to restore fertility, but not to the levels exhibited by control fish. Improved hormone therapy protocols need to be developed. With robust control over reproduction validated through multiple generations, the long-term risk of genetic introgression into wild populations would be negligible, as fertile escapees would be absent. Completely overcoming this problem will solidify the safety of transgenesis and gene editing and greatly aid in the acceptance, approval, and application of these catfishes for the benefit of the entire value chain, including the hatchery, the producer, the processor, and the consumer. Eventually, genetic biotechnology will be key to making aquaculture sustainable, efficient, and profitable while feeding the world.

## Figures and Tables

**Table 1:** Summary of transgenic research in catfishes, including fish species, delivery method, target genes, potential benefits, founder generation, integration efficiency percentage, germline transmission rate, sample size, validation of phenotype, and pleiotropic effects.

Species	Delivery Method	Target Gene(s)	Potential Benefits	Founder Generation	Integration Efficiency in P_1_ (%)	Germline Transmission Rate (%)	Sample Size	Phenotype Validated	Pleiotropic Effects	Reference
Channel catfish *Ictalurus punctatus*	Microinjection	Metallothionein-human growth hormone fusion gene (MThGH)	Growth enhancement	P_1_	NR	NR	NR	No	Reduced embryo survival	[51]
	Microinjection	Metallothionein-human growth hormone gene Rouse sarcoma virus (RSV)-rainbow trout growth hormone RSV-coho salmon growth hormone Rainbow trout vitellogenin gene	Growth enhancement	P_1_	2.1–15.2	NR	NR	No	Reduced embryo survival	[64]
	Microinjection	Salmonid growth hormone genes RSVLTR-rtGH1 cDNA and RSVLTR-csGH cDNA	Growth enhancement	P_1_	100	0–52	10 (P_1_)	Yes (26% improvement in growth in F_1_)	Deformities (13.6%) in P_1_	[65]
	Electroporation	Cecropin B gene (cec) from the moth *Hyalophora cecropia*, driven by the cytomegalovirus (CMV) promoter	Disease resistance	P_1_	NR	20.2–30.7	182 (F_1_)	Yes (Improved resistance to *F. columnare* and *E. ictaluri* in F_1_)	NR	[28]
	Electroporation	*nanos*, *vasa*, and *dead end*	Reproductive confinement	P_1_	NR	NR	393 (P_1_)	Yes (reduced sexual maturity	NR	[66]
	Electroporation	Masou salmon D5-desaturase	Body composition (n-3 fatty acids)	F_1_	NR	NR	37	Yes (33% increase in n-3 fatty acids)	Enhanced growth and resistance to *F. columnare*, lower tolerance to lower dissolved oxygen in some families	[67]
African catfish *Clarias gariepinus*	Electroporation	DNA plasmid (general construct)	Reporter/technical validation	P_1_	3.5	NR	212	No	NR	[68]
	Electroporation	DNA plasmid (general construct)	Reporter/technical validation	P_1_	22–47.5 (transgene expression)	NR	158	No	Abnormal development	[69]
	Microinjection	Plasmids: beta-galactosidase (*lacZ*) or luciferase (*luc*) with or without the CMV promoter/enhancer	Reporter/technical validation	P_1_	Transient expression	NR		No	NR	[70]
	Microinjection	Human coagulation factor VII (hFVII)	Bio-factory (Expression of human coagulation factor FVII)	P_1_	Transgene expression	NR	510	Blood clotting activity	NR	[71]
	Electroporation	Striped catfish (*Pangasianodon hypophthalmus*) growth hormone gene	Growth	P_1_	50	NR	6	No	NR	[72]
Striped catfish *Pangasianodon hypophthalmus*	Electroporation	Striped catfish (*Pangasianodon hypophthalmus*) growth hormone gene	Reporter/technical validation	P_1_	NR	NR	NR	No	NR	[73]
Mutiara catfish *Clarias gariepinus*	Electroporation	African catfish (*Clarias gariepinus*) growth hormone gene	Growth enhancement	P_1_, F_1_	NR	NR	NR	Yes	Improved feed conversion ratio	[74]
	Spawning 4th generation	African catfish (*Clarias gariepinus*) growth hormone gene	Growth enhancement	F_4_	-	92	260 (F_5_)	Yes	Increased ovary growth	[75]
Silver catfish *Rhamdia quelen*	Electroporation	Enhanced green fluorescent protein (EGFP)	Reporter/technical validation	P_1_	5–60	NR	120	Yes (EGFP expression)	NR	[76]
Yellow catfish *Tachysurus fulvidraco*	Microinjection	Enhanced yellow fluorescent protein (eYFP)	Reporter/technical validation	P_1_	0.43	NR	71	Yes (eYFP expression)	NR	[77]
	Microinjection	Growth hormone	Growth	P_1_	13.3	NR	301	NR	NR	[78]
	Microinjection	PDZ domain-containing gene (*pfpdz1*)	Male differentiation	P_1_	11.32	NR	53	Yes (partial female to male sex reversal)	NR	[79]

NR: not reported.

**Table 2:** Comparison of genetic modification approaches applied or proposed in catfish research.

Item	Traditional Transgenesis	Gene Knockout (NHEJ)	CRISPR-Assisted Insertion (Targeted Transgenesis)	Targeted Knock-In (HDR—No Foreign DNA)	Multiplex Gene Editing
Foreign DNA introduced	Yes—typically from another species	No	Yes—foreign gene at defined locus	No—endogenous sequence modification or correction	Can be multiple gene knockout, insertion, or a combination of both
Integration site control	Random, uncontrolled	N/A—no integration	Targeted to specific locus	Targeted to specific locus	Multiple defined loci simultaneously
Primary mechanism	DNA injected/electroporated into embryo → integrates randomly	Cas9 DSB repaired by error-prone NHEJ → indel → gene disruption	Cas9 DSB at target locus + HDR with foreign donor template	Cas9 DSB + HDR with endogenous-sequence donor → sequence replacement	Multiple sgRNAs with/without foreign DNA → simultaneous knockouts, insertions, or both
DSB required	No	Yes	Yes	Yes	Yes
HDR template required	No	No	Yes—donor with homology arms	Yes—donor with homology arms	Depends on components
Off-target risk	Insertional mutagenesis at random sites, position effects	Cas9 off-target indels	Cas9 off-target cuts + incomplete or mis-targeted HDR	Cas9 off-target cuts + incomplete repair or donor integration at off-target sites	Cumulative off-target risk + possibility of large chromosomal deletion from multiple sgRNA use
US regulatory classification (fish)	FDA pre-market approval required (NAD pathway)	FDA oversight required	FDA pre-market approval required (transgenic)	May qualify for lower regulatory tier under FDA guidance	Case-by-case
EU regulatory classification	Regulated as GMO	Regulated as GMO	Regulated as GMO	Regulated as GMO	Regulated as GMO
Environmental risk	Moderate to high	Lower—no foreign DNA introduced	Moderate	Lower—no foreign DNA introduced	Depends on combination
Consumer acceptance	Low—foreign DNA from other species	Higher than transgenesis—no foreign DNA; change is a loss of function; similar to natural mutation	Low—still transgenic; though more controlled than traditional transgenesis	Higher—no foreign DNA; modification is a correction or subtle change; similar to natural variation	Low to moderate depending on foreign DNA insertion
Commercial readiness in catfish	Limited—research lines established; no market approval in catfish to date	Research stage	Research stage	Not demonstrated in catfish	Research stage
Key catfish examples	Growth hormone-transgenic catfish [65,87]; cecropin B transgenic catfish [28].	*mstn*-knockout catfish [38,105]; *mc4r*-knockout catfish [106,107]	*cath*-transgenic channel catfish [108]	None reported in catfish	Insertion of *cath* and *cec* transgenes with simultaneous knockout of *lh*, *mc4r* and *mstn* [109]

DSB: double-strand break; FDA: Food and Drug Administration; GMO: genetically modified organism; HDR: homology-directed repair; NAD: new animal drug; NHEJ: non-homologous end joining.

**Table 3:** Comparison of genome-editing tools: action, structure, target-site recognition of CRISPR/Cas9, TALENs, ZFNs, and meganucleases.

Item		CRISPR/Cas9	TALENs	ZFNs	Mega Nucleases
Action	Recognition	RNA-DNA	Protein-DNA	Protein-DNA	Protein-DNA
	Mode of action	Double-strand breaks or single-strand nicks in the target DNA	Double-strand breaks in the target DNA	Double-strand breaks in the target DNA	Double-strand breaks.
	Off-target effects	High-fidelity variants (like SpCas9-HF1) have lower potential off-target effects than TALENs, ZFNs, and meganuclease	More observed off-target effects than CRISPR/Cas9	More potential off-target effects than TALENs	Potential off-target effects
Structure and construction	Components	sgRNA and SpCas9 protein	TALE DNA-binding domains and nonspecific FokI nuclease domain	Zinc finger domains Nonspecific FokI nuclease domain	Homodimers of two identical protein chains (LAGLIDADG scaffold family)
	Structural protein	Monomeric protein	Dimeric protein	Dimeric protein	Dimeric protein
	Catalytic domain	RuvC and HNH	Restriction endonuclease FokI	Restriction endonuclease FokI	Embedded within the structural protein (not separate)
	Multiplexing	Capable and easy	Labor-intensive and rarely used	Labor-intensive and rarely used	Labor-intensive and rarely used
Target sequence	Length (bp)	20–22	24–59	24–36	14–40
	Site of the target sequence	2–3 nucleotides adjacent to the protospacer adjacent motif (PAM)	Each TAL repeat binds to a base pair of DNA. Sequences targeted by TAL effector repeats are typically directly preceded by a thymine (T) that is required for maximal activity.	Each zinc finger binds a 3-bp DNA target. Not all 3-bp sequences can be targeted. Assembly of 3–4 zinc finger modules is required for specific recognition.	Only pre-existing mega nuclease recognition site. The absence of that same site from the targeting vector is required.

**Table 4:** Applications of CRISPR/Cas, TALENs, ZFNs, and meganucleases in catfish and other teleost research.

Tool	Application	Evidence in Catfish	Evidence in Other Teleosts	Catfish References	Other Teleost References
CRISPR/Cas9	Gene knockout	Yes	Yes	[38,107,119]	[40,120,121,122,123,124,125,126]
	Gene knock-in	Yes	Yes	[44,127]	[128,129,130]
	Base editing	No	Yes	Proposed future research	[131,132]
	Prime editing	No	Yes	Proposed future research	[133,134]
	Epigenetic editing (CRISPRi and CRISPRa)	No	Yes	Proposed future research	[135,136,137,138]
CRISPR-Cas12a	Gene knockout	No	Yes	Proposed future research	[126,139]
	Gene knock-in	No	Yes	Proposed future research	[140]
CRISPR-Cas13	Gene knockdown	No	Yes	Proposed future research	[141,142,143]
TALENs	Gene knockout	Yes	Yes	[144,145]	[146,147]
	Gene knock-in	No	Yes	None	[129]
ZFNs	Gene knockout	Yes	Yes	[148,149]	[150,151,152]
	Gene knock-in	No	Yes	None	[153]
Meganucleases	Transgenesis	No	Yes	None	[112]

**Table 5:** Summary of gene-editing research in catfishes, including the tools, delivery method, target genes, founder generation, sample size, editing efficiency percentage, germline transmission rate, validation of phenotype, potential benefits, and pleiotropic effects.

Species	Tools	Delivery Method	Target Gene(s)	Founder Generation	Sample Size	Editing Efficiency (%)	Germline Transmission Rate (%)	Phenotype Validated	Potential Benefits	Pleiotropic Effects	References
Yellow catfish *Tachysurus fulvidraco*	ZFNs	Microinjection	Myostatin (*mstna*)	P_1_	577	0–2	22.6	No	Growth	NR	[149]
	ZFNs	Microinjection	Myostatin (*mstna*)	F_1_	40	NR	25–50	Yes	Growth enhancement	NR	[39]
	TALENs	Microinjection	Myostatin (*mstnb*)	P_1_	4	2.2–14.6	0–16.4	No	No growth benefits	NR	[144]
	CRISPR/Cas9	Microinjection	*T. fulvidraco* PDZ domain (*pfpdz1*)	P_1_	150	0–27.6	NR	Yes (partial male-to-female sex reversal)	Male differentiation	NR	[79]
Southern catfish *Silurus meridionalis*	CRISPR/Cas9	Microinjection	cytochrome P450, family 26, subfamily A, polypeptide 1 (*cyp26a1*)	P_1_	20 (pooled)	34–90	NR	Yes	Functional genomics/meiosis regulation	NR	[155]
Channel catfish *Ictalurus punctatus*	ZFNs	Electroporation	Luteinizing hormone (*lh*)	P_1_	66	19.7	NR	Yes	Reproductive confinement	NR	[148]
	TALENs	Electroporation	Gonadotropin-releasing hormone (*gnrh*)	P_1_	51	52.9	NR	Yes	Reproductive confinement	NR	[145]
	TALENs	Electroporation	Follicle-stimulating hormone (*fsh*)	P_1_	57	63.2	50.3	Yes	Reproductive confinement		[156]
	CRISPR/Cas9	Microinjection	Myostatin (*mstnb*)	P_1_	330	88–100	NR	Yes	Growth enhancement	NR	[38]
	CRISPR/Cas9	Microinjection	Myostatin (*mstnb*)	P_1_	209	31–100	83–91	Yes	Growth enhancement	Enhanced disease resistance	[157]
	CRISPR/Cas9	Microinjection	Toll/interleukin 1 receptor domain-containing adapter molecule (*ticam1*) and rhamnose-binding lectin (*rbl*)	P_1_	62	79–88	NR	No	Disease resistance	NR	[118]
	CRISPR/Cas9	Microinjection	*ticam1* and *rbl*	P_1_	100	60–92	NR	No	Disease resistance	Congenital deformities	[158]
	CRISPR/Cas9	Microinjection	Knock-in (insertion of the alligator cathelicidin gene)	P_1_	414	3–29	NR	No	Disease resistance	Reduced fry survival	[108]
	CRISPR/Cas9	Microinjection	Knock-in (insertion of the masu salmon *elovl2* gene)	P_1_	216	37.5	NR	Yes (n-3 fatty acid content)	Body composition (n-3 fatty acid) Reproductive confinement	Morphological deformities	[45]
	CRISPR/Cas9	Electroporation	Knock-in (insertion of the masu salmon *elovl2* gene)	P_1_	21	19	NR	Yes (n-3 fatty acid content)	Body composition (n-3 fatty acid content)	NR	[159]
	CRISPR/Cas9	Microinjection	Knock-in (insertion of the masu salmon *elovl2* gene and rabbitfish *Δ4 fad* and *Δ6 fad* genes)	P_1_	558	0–25	NR	No	Body composition (n-3 fatty acid content)	NR	[160]
	CRISPR/Cas9	Microinjection	Knock-in (insertion of the *C. elegans fat-1* and *fat-2* genes)	P_1_	605	7.8–28.1	NR	Yes	Body composition	NR	[46]
	CRISPR/Cas9	Electroporation	Melanocortin-4 receptor (*mc4r*) gene	P_1_	129	30.6	NR	Yes	Growth enhancement	NR	[107]
	CRISPR/Cas9	Microinjection	Melanocortin-4 receptor (*mc4r*) gene	P_1_	411	66.7–90.6	NR	Yes	Growth enhancement	NR	[107]
	CRISPR/Cas9	Microinjection	Melanocortin-4 receptor (*mc4r*) gene	P_1_	18	33	71	Yes	Growth enhancement	NR	[106]
	CRISPR/Cas9	Microinjection	Knock-in of the masu salmon elongase gene in the melanocortin-4 (*mc4r*)	P_1_	92	6–54	NR	Yes	Growth Body composition (n-3 fatty acid content)	NR	[127]
	CRISPR/Cas9	Microinjection	Knock-in (insertion of the alligator cathelicidin (*cath*) gene and cecropin (*cec*) gene)	P_1_ *cath*, F_3_ *cec*	12	51.4–56.7	17.6–54.8	Yes	Disease resistance against *E. ictaluri*	NR	[161]
	CRISPR/Cas9	Microinjection	Knock-in (insertion of the alligator cathelicidin gene)	P_1_	499	10.8–22.4	NR	Yes	Disease resistance against *F. covae* and *E. ictaluri* Reproductive confinement	NR	[43]
	CRISPR/Cas9	Microinjection	Knock-in (insertion of the alligator cathelicidin gene and cecropin (*cec*) gene)	P_1_	1004	10.8–23.0	NR	No	Disease resistance Growth Reproductive confinement	NR	[109]
Blue catfish *Ictalurus furcatus*	CRISPR/Cas9	Microinjection	Knock-in (insertion of the alligator cathelicidin gene)	P_1_	184	10.3–24.5	NR	Yes	Disease resistance to *F. covae*	None	[44]
Striped catfish *Pangasianodon hypophthalmus*	CRISPR/Cas9	Microinjection	Dead end “*dnd1*” genes related to primordial germ cell migration and survival.	P_1_	180	95.8–98.1	NR	Yes	Reproductive confinement	NR	[162]

NR: not reported.

**Table 6:** Summary of sterilization strategies for catfish, including the method, efficiency, reversibility, and potential uses in aquaculture.

Species	Strategy	Mechanism/Target	Efficiency	Reversibility	Potential Uses in Aquaculture	References
Channel catfish *Ictalurus punctatus*	RNAi (ds-shRNA targeting *nanos*, *dnd*) with salt-sensitive promoters	Knockdown of PGC genes via a Tet-Off-like system (NaCl-regulated)	High	Yes (salinity-dependent repression)	Limited (risk of unintended fertility in estuarine environments)	[213]
	RNAi with copper-sensitive promoters (*ctr3*/*mctr*)	RNAi targeting *nanos*, *dnd* regulated by copper sulfate	High	Yes (copper-regulated)	Limited (pleiotropic growth reduction)	[214]
	nanos cDNA overexpression	Disruption of PGC development via gene overexpression	Moderate (less consistent than RNAi)	Potentially inducible	Limited (variable success)	[213]
	*gad65* transgenesis (GnRH neuron disruption)	Alters GnRH neuron migration, affecting the reproductive axis	Moderate (reduced spawning; incomplete sterility)	Yes (fertility restored in ~66% with hormone therapy)	Moderate (inconsistent sterility across individuals)	[35]
	*bax* overexpression (pro-apoptotic gene)	Germ cell ablation via apoptosis	Low–moderate (ineffective in females; better in males/F_1_)	No effective repression	Low (impractical repression system/doxycycline ineffective)	[36]
	ZFN-mediated knockout (*lh*)	Permanent disruption of the reproductive hormone gene	High (underdeveloped gonads; sterility)	No	Moderate (effective but inflexible)	[148]
	TALEN knockout (*cfgnrh*)	Targeted mutagenesis of the *gnrh* gene	Moderate–high (52.9% mutation; spawning decreased to 20–50%)	No	Moderate to low (delivery challenges and complexity)	[145]
	TALEN knockout (*fsh*) + hormone therapy	Gonadotropin gene disruption with hormonal therapy	Moderate (33–40% spawning untreated → ~80–89% with hormones)	Yes (hormone-induced recovery)	Moderate to high (controllable but requires intervention)	[156]
	CRISPR/Cas9 multi-trait editing (*lh* + antimicrobial gene)	Gene knockout + transgene insertion (sterility + disease resistance)	High (sterile unless hormonally induced)	Yes (hormone-dependent)	High (multifunctional and scalable)	[180,215]
Striped catfish *Pangasianodon hypophthalmus*	CRISPR/Cas9 *dnd1* knockout	Disruption of the germ cell development gene (*dnd1*)	High (impaired gonadal development)	No (permanent knockout)	High (effective genetic sterilization candidate)	[162]

## Data Availability

No new data were created or analyzed in this study. Data sharing is not applicable to this article.

## References

[1] Duarte C.M., Marbá N., Holmer M. (2007). Rapid domestication of marine species. Science.

[2] Ferraris C.J. (2007). Checklist of catfishes, recent and fossil (Osteichthyes: Siluriformes), and catalogue of siluriform primary types. Zootaxa.

[3] Nelson J.S., Grande T.C., Wilson M.V. (2016). Fishes of the World.

[4] USDA 2023 Census of Aquaculture. https://www.nass.usda.gov/Publications/AgCensus/2022/Online_Resources/Aquaculture/Aqua.pdf.

[5] Dunham R.A., Elaswad A. (2018). Catfish biology and farming. Annu. Rev. Anim. Biosci..

[6] Tave D., McGinty A.S., Chappell J.A., Smitherman R. (1981). Relative harvestability by angling of blue catfish, channel catfish, and their reciprocal hybrids. Am. J. Fish. Manag..

[7] Arias C.R., Cai W., Peatman E., Bullard S.A. (2012). Catfish hybrid *Ictalurus punctatus* × *I. furcatus* exhibits higher resistance to *Columnaris* disease than the parental species. Dis. Aquat. Org..

[8] Bosworth B.G. (2012). Effects of winter feeding on growth, body composition, and processing traits of co-cultured blue catfish, channel catfish, and channel catfish×blue catfish hybrids. N. Am. J. Aquac..

[9] Bosworth B.G., Wolters W.R., Silva J.L., Chamul R.S., Park S. (2004). Comparison of production, meat yield, and meat quality traits of NWAC103 line channel catfish, Norris line channel catfish, and female channel catfish× male blue catfish F1 hybrids. N. Am. J. Aquac..

[10] Giudice J.J. (1966). Growth of a blue x channel catfish hybrid as compared to its parent species. Prog. Fish-Cult..

[11] Elaswad A., Khalil K., Ye Z., Alsaqufi A., Abdelrahman H., Su B., Perera D.A., Dong S., Abass N., Dunham R. (2019). Effects of cecropin transgenesis and interspecific hybridization on the resistance to *Ichthyophthirius multifiliis* in channel catfish and female channel catfish× male blue catfish hybrids. N. Am. J. Aquac..

[12] Argue B.J., Liu Z., Dunham R.A. (2003). Dress-out and fillet yields of channel catfish, *Ictalurus punctatus*, blue catfish, *Ictalurus furcatus*, and their F1, F2 and backcross hybrids. Aquaculture.

[13] Dunham R.A., Smitherman R.O., Webber C. (1983). Relative tolerance of channel x blue hybrid and channel catfish to low oxygen concentrations. Prog. Fish-Cult..

[14] Dunham R.A., Smitherman R.O., Goodman R.K., Kemp P. (1986). Comparison of strains, crossbreeds and hybrids of channel catfish for vulnerability to angling. Aquaculture.

[15] Dunham R. (2023). Aquaculture and Fisheries Biotechnology: Genetic Approaches.

[16] Hanson T., Sites D. (2014). U.S. Catfish Database. Mississippi State University, Agricultural Economics Information Report.

[17] Hanson T.R., Bott L.B., Chappell J.A., Whitis G.N., Kelly A.M., Roy L.A. (2020). Research verification of single- and multiple-batch production practices at two channel catfish farms in Western Alabama. N. Am. J. Aquac..

[18] Engle C.R., Christie T.W., Dorr B.S., Kumar G., Davis B., Roy L.A., Kelly A.M. (2021). Principal economic effects of cormorant predation on catfish farms. J. World Aquac. Soc..

[19] Brown T.W., Chappell J.A., Boyd C.E. (2011). A commercial-scale, in-pond raceway system for Ictalurid catfish production. Aquac. Eng..

[20] Cheatham M., Kumar G., Tucker C., Rutland B. (2023). Research verification of four commercial scale split-pond designs. Aquac. Eng..

[21] FAO (2024). The State of World Fisheries and Aquaculture 2024 Blue Transformation in Action.

[22] Ogunji J., Wuertz S. (2023). Aquaculture development in Nigeria: The second biggest aquaculture producer in Africa. Water.

[23] Opiyo M.A., Marijani E., Muendo P., Odede R., Leschen W., Charo-Karisa H. (2018). A Review of aquaculture production and health management practices of farmed fish in Kenya. Int. J. Vet. Sci. Med..

[24] Gustiano R., Prakoso V., Radona D., Dewi R.S., Saputra A., Nurhidayat (2021). A sustainable aquaculture model in Indonesia: Multi-biotechnical approach in *Clarias* farming. IOP Conf. Ser. Earth Environ. Sci..

[25] Waltz E. (2017). First genetically engineered salmon sold in Canada. Nature.

[26] AquaBounty AquaBounty Announces Plans to Cease Fish Farming Operations. https://investors.aquabounty.com/news-releases/news-release-details/aquabounty-announces-plans-cease-fish-farming-operations.

[27] Du S.J., Gong Z., Fletcher G.L., Shears M.A., King M.J., Idler D.R., Hew C.L. (1992). Growth enhancement in transgenic Atlantic salmon by the use of an “all fish” chimeric growth hormone gene construct. Nat. Biotechnol..

[28] Dunham R.A., Warr G.W., Nichols A., Duncan P.L., Argue B., Middleton D., Kucuktas H. (2002). Enhanced bacterial disease resistance of transgenic channel catfish *Ictalurus punctatus* possessing cecropin genes. Mar. Biotechnol..

[29] Dunham R.A. (2009). Transgenic fish resistant to infectious diseases, their risk and prevention of escape into the environment and future candidate genes for disease transgene manipulation. Comp. Immunol. Microbiol. Infect. Dis..

[30] Wang R., Zhang P., Gong Z., Hew C. (1995). Expression of the antifreeze protein gene in transgenic goldfish (*Carassius auratus*) and its implication in cold adaptation. Mol. Mar. Biol. Biotechnol..

[31] Gong Z., Wan H., Tay T.L., Wang H., Chen M., Yan T. (2003). Development of transgenic fish for ornamental and bioreactor by strong expression of fluorescent proteins in the skeletal muscle. Biochem. Biophys. Res. Commun..

[32] Morita T., Yoshizaki G., Kobayashi M., Watabe S., Takeuchi T. (2004). Fish eggs as bioreactors: The production of bioactive luteinizing hormone in transgenic trout embryos. Transgenic Res..

[33] Cachot J., Law M., Pottier D., Peluhet L., Norris M., Budzinski H., Winn R. (2007). Characterization of toxic effects of sediment-associated organic pollutants using the λ transgenic medaka. Environ. Sci. Technol..

[34] Cheng Q., Su B., Qin Z., Weng C.-C., Yin F., Zhou Y., Fobes M., Perera D.A., Shang M., Soller F. (2014). Interaction of diet and the masou salmon Δ5-desaturase transgene on Δ6-desaturase and stearoyl-CoA desaturase gene expression and N-3 fatty acid level in common carp (*Cyprinus carpio*). Transgenic Res..

[35] Ye Z., Elaswad A., Su B., Alsaqufi A., Shang M., Bugg W.S., Qin G., Drescher D., Li H., Qin Z. (2024). Reversible sterilization of channel catfish via overexpression of glutamic acid decarboxylase gene. Animals.

[36] Ye Z., Elaswad A., Qin G., Zhang D., Su B., Khalil K., Qin Z., Abass N.Y., Cheng Q., Odin R. (2025). Sterilization of channel catfish (*Ictalurus punctatus*) via overexpression of *bax* gene regulated by a tet-off system in the primordial germ cells. Mar. Biotechnol..

[37] Xu C., Wu G., Zohar Y., Du S.-J. (2003). Analysis of myostatin gene structure, expression and function in zebrafish. J. Exp. Biol..

[38] Khalil K., Elayat M., Khalifa E., Daghash S., Elaswad A., Miller M., Abdelrahman H., Ye Z., Odin R., Drescher D. (2017). Generation of myostatin gene-edited channel catfish (*Ictalurus punctatus*) via zygote injection of CRISPR/Cas9 system. Sci. Rep..

[39] Zhang X., Wang F., Dong Z., Dong X., Chi J., Chen H., Zhao Q., Li K. (2020). A new strain of yellow catfish carrying genome edited myostatin alleles exhibits double muscling phenotype with hyperplasia. Aquaculture.

[40] Tao B., Tan J., Chen L., Xu Y., Liao X., Li Y., Chen J., Song Y., Hu W. (2021). CRISPR/Cas9 system-based myostatin-targeted disruption promotes somatic growth and adipogenesis in loach, *Misgurnus anguillicaudatus*. Aquaculture.

[41] Wu Y., Wu T., Yang L., Su Y., Zhao C., Li L., Cai J., Dai X., Wang D., Zhou L. (2023). Generation of fast growth Nile tilapia (*Oreochromis niloticus*) by myostatin gene mutation. Aquaculture.

[42] Ou M., Wang F., Li K., Wu Y., Huang S., Luo Q., Liu H., Zhang X., Fei S., Chen K. (2023). Generation of myostatin gene-edited blotched snakehead (*Channa maculata*) using CRISPR/Cas9 system. Aquaculture.

[43] Wang J., Su B., Xing D., Bruce T.J., Li S., Bern L., Shang M., Johnson A., Simora R.M.C., Coogan M. (2024). Generation of eco-friendly and disease-resistant channel catfish (*Ictalurus punctatus*) harboring the alligator cathelicidin gene via CRISPR/Cas9 engineering. Engineering.

[44] Wang J., Su B., Al-Armanazi J., Wise A.L., Shang M., Bern L., Li S., Xing D., Johnson A., Wang W. (2023). Integration of alligator cathelicidin gene via two CRISPR/Cas9-assisted systems enhances bacterial resistance in blue catfish, *Ictalurus furcatus*. Aquaculture.

[45] Xing D., Su B., Li S., Bangs M., Creamer D., Coogan M., Wang J., Simora R., Ma X., Hettiarachchi D. (2022). CRISPR/Cas9-mediated transgenesis of the masu salmon (*Oncorhynchus masou*) elovl2 gene improves n-3 fatty acid content in channel catfish (*Ictalurus punctatus*). Mar. Biotechnol..

[46] Xing D., Shang M., Li S., Wang W., Hasin T., Hettiarachchi D., Alston V., Bern L., Qin Z., Su B. (2023). CRISPR/Cas9-mediated precision integration of fat-1 and fat-2 from *Caenorhabditis elegans* at long repeated sequence in channel catfish (*Ictalurus punctatus*) and the impact on n-3 fatty acid level. Aquaculture.

[47] Palmiter R.D., Brinster R.L., Hammer R.E., Trumbauer M.E., Rosenfeld M.G., Birnberg N.C., Evans R.M. (1982). Dramatic growth of mice that develop from eggs microinjected with metallothionein–growth hormone fusion genes. Nature.

[48] Zhu Z., He L., Chen S. (1985). Novel gene transfer into the fertilized eggs of gold fish (*Carassius auratus* L. 1758). J. Appl. Ichthyol..

[49] Chourrout D., Guyomard R., Houdebine L.-M. (1986). High efficiency gene transfer in rainbow trout *(Salmo gairdneri* Rich.) by microinjection into egg cytoplasm. Aquaculture.

[50] Zhu Z., Xu K., Li G., Xie Y., He L. (1986). Biological effects of human growth hormone gene microinjected into the fertilized eggs of loach *Misgurnus anguillicaudatus* (Cantor). Chin. Sci. Bull..

[51] Dunham R.A., Eash J., Askins J., Townes T.M. (1987). Transfer of the metallothionein-human growth hormone fusion gene into channel catfish. Trans. Am. Fish. Soc..

[52] Powers D.A., Hereford L., Cole T., Chen T.T., Lin C., Kight K., Creech K., Dunham R. (1992). Electroporation: A method for transferring genes into the gametes of zebrafish (*Brachydanio rerio*), channel catfish (*Ictalurus punctatus*), and common carp (*Cyprinus carpio*). Mol. Mar. Biol. Biotechnol..

[53] Fletcher G.L., Shears M.A., King M.J., Davies P.L., Hew C.L. (1988). Evidence for antifreeze protein gene transfer in Atlantic salmon (*Salmo salar*). Can. J. Fish. Aquat. Sci..

[54] Stuart G.W., McMurray J.V., Westerfield M. (1988). Replication, integration and stable germ-line transmission of foreign sequences injected into early zebrafish embryos. Development.

[55] Inoue K., Yamashita S., Hata J.-I., Kabeno S., Asada S., Nagahisa E., Fujita T. (1990). Electroporation as a new technique for producing transgenic fish. Cell Differ. Dev..

[56] Zhang P., Hayat M., Joyce C., Gonzalez-Villaseñor L.I., Lin C., Dunham R.A., Chen T.T., Powers D.A. (1990). Gene transfer, expression and inheritance of pRSV-rainbow trout-GH cDNA in the common carp, *Cyprinus carpio* (Linnaeus). Mol. Reprod. Dev..

[57] Chen T.T., Kight K., Lin C., Powers D.A., Hayat M., Chatakondi N., Ramboux A.C., Duncan P.L., Dunham R.A. (1993). Expression and inheritance of RSVLTR-rtGH1 complementary DNA in the transgenic common carp, *Cyprinus carpio*. Mol. Mar. Biol. Biotechnol..

[58] Rahman M.A., Maclean N. (1992). Production of transgenic tilapia (*Oreochromis niloticus*) by one-cell-stage microinjection. Aquaculture.

[59] Martínez R., Estrada M.P., Berlanga J., Guillén I., Hernández O., Cabrera E., Pimentel R., Morales R., Herrera F., Morales A. (1996). Growth enhancement in transgenic tilapia by ectopic expression of tilapia growth hormone. Mol. Mar. Biol. Biotechnol..

[60] Gross M.L., Schneider J.F., Moav N., Moav B., Alvarez C., Myster S.H., Liu Z., Hallerman E.M., Hackett P.B., Guise K.S. (1992). Molecular analysis and growth evaluation of northern pike (*Esox lucius*) microinjected with growth hormone genes. Aquaculture.

[61] Devlin R.H., Yesaki T.Y., Donaldson E.M., Du S.J., Hew C.-L. (1995). Production of germline transgenic Pacific salmonids with dramatically increased growth performance. Can. J. Fish. Aquat. Sci..

[62] Pitkänen T.I., Krasnov A., Teerijoki H., Mölsä H. (1999). Transfer of growth hormone (GH) transgenes into Arctic charr (*Salvelinus alpinus* L.): I. Growth response to various GH constructs. Genet. Anal. Biomol. Eng..

[63] Nam Y.K., Noh J.K., Cho Y.S., Cho H.J., Cho K.-N., Kim C.G., Kim D.S. (2001). Dramatically accelerated growth and extraordinary gigantism of transgenic mud loach *Misgurnus mizolepis*. Transgenic Res..

[64] Hayat M., Joyce C.P., Townes T.M., Chen T.T., Powers D.A., Dunham R.A. (1991). Survival and integration rate of channel catfish and common carp embryos microinjected with DNA at various developmental stages. Aquaculture.

[65] Dunham R., Ramboux A., Duncan P., Hayat M., Chen T., Lin C., Kight K., Gonzalez-Villasenor I., Powers D. (1992). Transfer, expression, and inheritance of salmonid growth hormone genes in channel catfish, *Ictalurus punctatus*, and effects on performance traits. Mol. Mar. Biol. Biotechnol..

[66] Su B., Shang M., Grewe P.M., Patil J.G., Peatman E., Perera D.A., Cheng Q., Li C., Weng C.-C., Li P. (2015). Suppression and restoration of primordial germ cell marker gene expression in channel catfish, *Ictalurus punctatus*, using knockdown constructs regulated by copper transport protein gene promoters: Potential for reversible transgenic sterilization. Theriogenology.

[67] Huang Y., Bugg W., Bangs M., Qin G., Drescher D., Backenstose N., Weng C.C., Zhang Y., Khalil K., Dong S. (2021). Direct and pleiotropic effects of the masou salmon delta-5 desaturase transgene in F1 channel catfish (*Ictalurus punctatus*). Transgenic Res..

[68] Müller F., Ivics Z., Erdélyi F., Papp T., Váradi L., Horváth L., Maclean N., Orbán L. (1992). Introducing foreign genes into fish eggs with electroporated sperm as a carrier. Mol. Mar. Biol. Biotechnol..

[69] Müller F., Lele Z., Váradi L., Menczel L., Orbán L. (1993). Efficient transient expression system based on square pulse electroporation and in vivo luciferase assay of fertilized fish eggs. FEBS Lett..

[70] Volckaert F.A., Hellemans B.A., Galbusera P., Ollevier F., Sekkali B., Belayew A. (1994). Replication, expression, and fate of foreign DNA during embryonic and larval development of the African catfish (*Clarias gariepinus*). Mol. Mar. Biol. Biotechnol..

[71] Hwang G., Müller F., Rahman M.A., Williams D.W., Murdock P.J., Pasi K.J., Goldspink G., Farahmand H., Maclean N. (2004). Fish as bioreactors: Transgene expression of human coagulation factor VII in fish embryos. Mar. Biotechnol..

[72] Marnis H., Dewi R.R.S.P.S., Imron I., Iswanto B. (2012). Distribution and expression of striped catfish (*Pangasionodon hypophtalmus*) growth hormone gene (PhGH) in the organ of African catfish (*Clarias gariepinus*) transgenic founder. Indones. Aquac. J..

[73] Dewi R.R.S.P.S., Alimuddin A., Sudrajat A.O., Sumantadinata K., Sularto S. (2010). Optimal electroporation condition for sperm mediated gene transfer in stripped catfish (*Pangasionodon hypophthalmus*). Indones. Aquac. J..

[74] Iskandar I., Buwono I., Agung M. (2018). The growth performance of F1 transgenic mutiara catfish. IOP Conf. Ser. Earth Environ. Sci..

[75] Grandiosa R., Buwono I.D., Mulyani Y., Pratiwy F.M. (2026). Impact of temperature upon expression levels of GnRHr, THRr, and FSHr leading to gonadal maturation of G5 transgenic Mutiara strain female catfish (*Clarias gariepinus*). Agric. Res..

[76] Collares T., Campos V.F., Seixas F.K., Cavalcanti P.V., Dellagostin O.A., Moreira H.L.M., Deschamps J.C. (2010). Transgene transmission in South American catfish (*Rhamdia quelen*) larvae by sperm-mediated gene transfer. J. Biosci..

[77] Ge J., Dong Z., Li J., Xu Z., Song W., Bao J., Liang D., Li J., Li K., Jia W. (2012). Isolation of yellow catfish β-actin promoter and generation of transgenic yellow catfish expressing enhanced yellow fluorescent protein. Transgenic Res..

[78] Ge J., Song W., Dong Z. (2013). Generation of “all fish” growth hormone gene transgenic yellow catfish founders. J. Nanjing Univ. Nat. Sci..

[79] Dan C., Lin Q., Gong G., Yang T., Xiong S., Xiong Y., Huang P., Gui J.-F., Mei J. (2018). A novel PDZ domain-containing gene is essential for male sex differentiation and maintenance in yellow catfish (*Pelteobagrus fulvidraco*). Sci. Bull..

[80] Rahman M., Ronyai A., Engidaw B., Jauncey K., Hwang G.L., Smith A., Roderick E., Penman D., Varadi L., Maclean N. (2001). Growth and nutritional trials on transgenic Nile tilapia containing an exogenous fish growth hormone gene. J. Fish Biol..

[81] Johnsson J.I., Björnsson B.T. (1994). Growth hormone increases growth rate, appetite and dominance in juvenile rainbow trout, *Oncorhynchus mykiss*. Anim. Behav..

[82] Abass N.Y., Su B., Perera D.A., Qin Z., Li H., Alsaqufi A., Elaswad A., Ye Z., Dong S., Dunham R.A. (2021). Effects of family and promoter on growth performance of ccGH cDNA transgenic channel catfish, *Ictalurus punctatus*, grown in a trough culture system. Aquaculture.

[83] Abass N.Y., Su B., Alsaqufi A., Elaswad A., Qin Z., Li H., Odin R., Ye Z., Dunham R.A. (2021). Growth differences of growth hormone transgenic female and male channel catfish, *Ictalurus punctatus*, grown in earthen ponds to sexual maturation. Mar. Biotechnol..

[84] Celino-Brady F.T., Breves J.P., Seale A.P. (2022). Sex-specific responses to growth hormone and luteinizing hormone in a model teleost, the Mozambique tilapia. Gen. Comp. Endocrinol..

[85] Chan M.T.T., Muttray A., Sakhrani D., Woodward K., Kim J.H., Christensen K.A., Koop B.F., Devlin R.H. (2021). Sexually dimorphic growth stimulation in a strain of growth hormone transgenic coho salmon (*Oncorhynchus kisutch*). Mar. Biotechnol..

[86] Lu J., Li J., Furuya Y., Yoshizaki G., Sun H., Endo M., Haga Y., Satoh S., Takeuchi T. (2009). Efficient productivity and lowered nitrogen and phosphorus discharge load from GH-transgenic tilapia (*Oreochromis niloticus*) under visual satiation feeding. Aquaculture.

[87] Buwono I.D., Junianto J., Iskandar I., Alimuddin A. (2019). Growth and expression level of growth hormone in transgenic mutiara catfish second generation. J. Biotech. Res..

[88] Buwono I.D., Iskandar I., Grandiosa R. (2021). Growth hormone transgenesis and feed composition influence growth and protein and amino acid content in transgenic G3 mutiara catfish (*Clarias gariepinus*). Aquac. Int..

[89] Buwono I.D., Iskandar I., Grandiosa R. (2023). CgGH and IGF-1 expression level and growth response of G4 transgenic mutiara strain catfish (*Clarias gariepinus*) reared at different stocking densities. Aquac. Int..

[90] Sarmasik A., Warr G., Chen T.T. (2002). Production of transgenic medaka with increased resistance to bacterial pathogens. Mar. Biotechnol..

[91] Zhong J., Wang Y., Zhu Z. (2002). Introduction of the human lactoferrin gene into grass carp (*Ctenopharyngodon idellus*) to increase resistance against GCH virus. Aquaculture.

[92] Hsieh J.-C., Pan C.-Y., Chen J.-Y. (2010). Tilapia hepcidin (TH) 2-3 as a transgene in transgenic fish enhances resistance to *Vibrio vulnificus* infection and causes variations in immune-related genes after infection by different bacterial species. Fish Shellfish Immunol..

[93] Peng K.-C., Pan C.-Y., Chou H.-N., Chen J.-Y. (2010). Using an improved Tol2 transposon system to produce transgenic zebrafish with epinecidin-1 which enhanced resistance to bacterial infection. Fish Shellfish Immunol..

[94] Fletcher G.L., Hobbs R.S., Evans R.P., Shears M.A., Hahn A.L., Hew C.L. (2011). Lysozyme transgenic Atlantic salmon (*Salmo salar* L.). Aquac. Res..

[95] Elaswad A., Dunham R. (2018). Disease reduction in aquaculture with genetic and genomic technology: Current and future approaches. Rev. Aquacult..

[96] Weifeng M., Yaping W., Wenbo W., Bo W., Jianxin F., Zuoyan Z. (2004). Enhanced resistance to *Aeromonas hydrophila* infection and enhanced phagocytic activities in human lactoferrin-transgenic grass carp (*Ctenopharyngodon idellus*). Aquaculture.

[97] Alimuddin, Yoshizaki G., Kiron V., Satoh S., Takeuchi T. (2005). Enhancement of EPA and DHA biosynthesis by over-expression of masu salmon Δ6-desaturase-like gene in zebrafish. Transgenic Res..

[98] Alimuddin, Yoshizaki G., Kiron V., Satoh S., Takeuchi T. (2007). Expression of masu salmon Δ5-desaturase-like gene elevated EPA and DHA biosynthesis in zebrafish. Mar. Biotechnol..

[99] Devlin R.H., Yesaki T.Y., Biagi C.A., Donaldson E.M., Swanson P., Chan W.-K. (1994). Extraordinary salmon growth. Nature.

[100] Maclean N., Rahman A., Maclean N. (1994). Transgenic fish. Animals with Novel Genes.

[101] Wang Y., Hu W., Wu G., Sun Y., Chen S., Zhang F., Zhu Z., Feng J., Zhang X. (2001). Genetic analysis of “all-fish” growth hormone gene transferred carp (*Cyprinus carpio* L.) and its F1 generation. Chin. Sci. Bull..

[102] Ishtiaq A., Xiong F., Pang S.-C., He M.-D., Waters M.J., Zhu Z.-Y., Sun Y.-H. (2011). Activation of GH signaling and GH-independent stimulation of growth in zebrafish by introduction of a constitutively activated GHR construct. Transgenic Res..

[103] Maclean N., Penman D., Zhu Z. (1987). Introduction of novel genes into fish. Nat. Biotechnol..

[104] Dobie K.W., Lee M., Fantes J.A., Graham E., Clark A.J., Springbett A., Lathe R., McClenaghan M. (1996). Variegated transgene expression in mouse mammary gland is determined by the transgene integration locus. Proc. Natl. Acad. Sci. USA.

[105] Zhang X., Wang F., Ou M., Liu H., Luo Q., Fei S., Zhao J., Chen K., Zhao Q., Li K. (2023). Effects of myostatin b knockout on offspring body length and skeleton in yellow catfish (*Pelteobagrus fulvidraco*). Biology.

[106] Coogan M., Alston V., Su B., Khalil K., Elaswad A., Khan M., Johnson A., Xing D., Li S., Wang J. (2022). Improved growth and high inheritance of melanocortin-4 receptor (*mc4r*) mutation in CRISPR/Cas-9 gene-edited channel catfish, *Ictalurus punctatus*. Mar. Biotechnol..

[107] Khalil K., Elaswad A., Abdelrahman H., Michel M., Chen W., Liu S., Odin R., Ye Z., Drescher D., Vo K. (2023). Editing the melanocortin-4 receptor gene in channel catfish using the CRISPR-Cas9 system. Fishes.

[108] Simora R.M.C., Xing D., Bangs M.R., Wang W., Ma X., Su B., Khan M.G., Qin Z., Lu C., Alston V. (2020). CRISPR/Cas9-mediated knock-in of alligator cathelicidin gene in a non-coding region of channel catfish genome. Sci. Rep..

[109] Wang J., Torres I.M., Shang M., Al-Armanazi J., Dilawar H., Hettiarachchi D.U., Paladines-Parrales A., Chambers B., Pottle K., Soman M. (2024). One-step knock-in of two antimicrobial peptide transgenes at multiple loci of catfish by CRISPR/Cas9-mediated multiplex genome engineering. Int. J. Biol. Macromol..

[110] Rembold M., Lahiri K., Foulkes N.S., Wittbrodt J. (2006). Transgenesis in fish: Efficient selection of transgenic fish by co-injection with a fluorescent reporter construct. Nat. Protoc..

[111] Soroldoni D., Hogan B.M., Oates A.C., Lieschke G., Oates A., Kawakami K. (2009). Simple and efficient transgenesis with meganuclease constructs in zebrafish. Zebrafish. Methods in Molecular Biology.

[112] Thermes V., Grabher C., Ristoratore F., Bourrat F., Choulika A., Wittbrodt J., Joly J.-S. (2002). I-SceI meganuclease mediates highly efficient transgenesis in fish. Mech. Dev..

[113] Urnov F.D., Miller J.C., Lee Y.-L., Beausejour C.M., Rock J.M., Augustus S., Jamieson A.C., Porteus M.H., Gregory P.D., Holmes M.C. (2005). Highly efficient endogenous human gene correction using designed zinc-finger nucleases. Nature.

[114] Mali P., Yang L., Esvelt K.M., Aach J., Guell M., DiCarlo J.E., Norville J.E., Church G.M. (2013). RNA-guided human genome engineering via Cas9. Science.

[115] Doudna J.A., Charpentier E. (2014). The new frontier of genome engineering with CRISPR-Cas9. Science.

[116] Ran F.A., Hsu P.D., Wright J., Agarwala V., Scott D.A., Zhang F. (2013). Genome engineering using the CRISPR-Cas9 system. Nat. Protoc..

[117] Varshney G.K., Pei W., LaFave M.C., Idol J., Xu L., Gallardo V., Carrington B., Bishop K., Jones M., Li M. (2015). High-throughput gene targeting and phenotyping in zebrafish using CRISPR/Cas9. Genome Res..

[118] Elaswad A., Khalil K., Cline D., Page-McCaw P., Chen W., Michel M., Cone R., Dunham R. (2018). Microinjection of CRISPR/Cas9 protein into channel catfish, *Ictalurus punctatus*, embryos for gene editing. J. Vis. Exp..

[119] Wang X., Xie Y., Lin Q., Xiong Y., Liu Y., Ge S., Tan Q., He Z., Jiang Y., Han Q. (2026). Genetic deletion of miR-200a/200b increases growth and feed conversion efficiency in yellow catfish. Sci. China Life Sci..

[120] Hwang W.Y., Fu Y., Reyon D., Maeder M.L., Tsai S.Q., Sander J.D., Peterson R.T., Yeh J.J., Joung J.K. (2013). Efficient in vivo genome editing using RNA-guided nucleases. Nat. Biotechnol..

[121] Yeh Y.-C., Kinoshita M., Ng T.H., Chang Y.-H., Maekawa S., Chiang Y.-A., Aoki T., Wang H.-C. (2017). Using CRISPR/Cas9-mediated gene editing to further explore growth and trade-off effects in myostatin-mutated F4 medaka (*Oryzias latipes*). Sci. Rep..

[122] Sun Y., Zheng G.-D., Nissa M., Chen J., Zou S.-M. (2020). Disruption of *mstna* and *mstnb* gene through CRISPR/Cas9 leads to elevated muscle mass in blunt snout bream (*Megalobrama amblycephala*). Aquaculture.

[123] Kuang Y., Zheng X., Cao D., Sun Z., Tong G., Xu H., Yan T., Tang S., Chen Z., Zhang T. (2023). Generate a new crucian carp (*Carassius auratus*) strain without intermuscular bones by knocking out *bmp6*. Aquaculture.

[124] Li M., Yang H., Zhao J., Fang L., Shi H., Li M., Sun Y., Zhang X., Jiang D., Zhou L. (2014). Efficient and heritable gene targeting in tilapia by CRISPR/Cas9. Genetics.

[125] Yang Z., Le W., Sun F., Wong J., Lee M., Yue G.H. (2026). CRISPR/Cas9-mediated *mstnb* knockout enhances muscle growth in salt-tolerant tilapia. Aquaculture.

[126] López-Porras A., Berg R.S., Burgerhout E., Hansen Ø.J., Györkei Á., Qiao S.-W., Johansen F.-E. (2024). CRISPR-Cas9/Cas12a-based genome editing in Atlantic cod (*Gadus morhua*). Aquaculture.

[127] Coogan M., Xing D., Su B., Alston V., Johnson A., Khan M., Khalil K., Elaswad A., Li S., Wang J. (2023). CRISPR/Cas9-mediated knock-in of masu salmon (*Oncorhyncus masou*) elongase gene in the melanocortin-4 (*mc4r*) coding region of channel catfish (*Ictalurus punctatus*) genome. Transgenic Res..

[128] Auer T.O., Duroure K., De Cian A., Concordet J.-P., Del Bene F. (2014). Highly efficient CRISPR/Cas9-mediated knock-in in zebrafish by homology-independent DNA repair. Genome Res..

[129] Zhang Y., Huang H., Zhang B., Lin S. (2016). TALEN-and CRISPR-enhanced DNA homologous recombination for gene editing in zebrafish. Method. Cell Biol..

[130] Albadri S., Del Bene F., Revenu C. (2017). Genome editing using CRISPR/Cas9-based knock-in approaches in zebrafish. Methods.

[131] Zhong Z., Hu X., Zhang R., Liu X., Chen W., Zhang S., Sun J., Zhong T.P. (2025). Improving precision base editing of the zebrafish genome by Rad51DBD-incorporated single-base editors. J. Genet. Genom..

[132] Zheng S., Liu Y., Xia X., Xiao J., Ma H., Yuan X., Zhang Y., Chen Z., Peng G., Li W. (2025). Sequence context-agnostic TadA-derived cytosine base editors for genome-wide editing in zebrafish. Adv. Sci..

[133] Ono Y., Peterka M., Love M., Khan A., Bowers F., Bhandari A., Gordon E., Ball J.S., Hammond C., Tyler C.R. (2026). Optimised genome editing for precise DNA insertion and substitution using prime editors in zebrafish. eLife.

[134] Vanhooydonck M., De Neef E., De Saffel H., Boel A., Willaert A., Callewaert B., Claes K.B. (2025). Prime editing outperforms homology-directed repair as a tool for CRISPR-mediated variant knock-in in zebrafish. Lab Anim..

[135] Huang S., Wu M., Huang Y., Wu Y., Xu J., Li S., Zhou L. (2026). Efficient epigenetic editing of the zebrafish *slc45a2* gene using the CRISPR/dCas9 system and tandem guide RNAs. Front. Mar. Sci..

[136] Liang M., Xu Z., Long L., Wang P., Chen Q., Shi G., Liang F., Zhao J., Mai K., Miao Y. (2026). Enhanced muscle growth via DNA methylation editing of *mstnb* exon in zebrafish. Aquac. Fish..

[137] Lin L., Zhang J.-J., Liu B.-H., Du S., Zhang Y.-Q., Yang Y., Li C., Dong C.-C., He Y.-B., Wang Q. (2026). Epigenetic editing of marine medaka (*Oryzias melastigma*) *fgf2* using CRISPR/dCas9-Tet1CD. Zool. Res..

[138] Shi M., Ge W., Li C., Liu B., Deng X., Liu C., Zheng M., Zhang P., Li L., Guo Y. (2026). Versatile CRISPR-Cas tools for gene regulation in zebrafish via an enhanced Q binary system. Adv. Sci..

[139] Li H., Liu L., Wang X., Zhang R., Zhu H. (2025). Enhancing genome editing efficiency in goldfish (*Carassius auratus*) through utilization of CRISPR-Cas12a (Cpf1) temperature dependency. Int. J. Biol. Macromol..

[140] Han B., Zhang Y., Zhou Y., Zhang B., Krueger C.J., Bi X., Zhu Z., Tong X., Zhang B. (2022). ErCas12a and T5exo-ErCas12a mediate simple and efficient genome editing in zebrafish. Biology.

[141] Nishimura T., Takahashi E., Fujimoto T. (2024). Sterilization of fish through adaptable gRNAs targeting dnd1 using CRISPR-Cas13d system. Aquaculture.

[142] Määttä K., Chen Y.-C., Pihlström S., Mäkitie R.E., Dambroise E., Legeai-Mallet L., Panula P., Mäkitie O., Pekkinen M. (2025). Utilizing CRISPR-Cas13d-knockdown in zebrafish to study a rare monogenic bone fragility syndrome. JBMR Plus.

[143] Ventura Fernandes B.H., Junqueira M.S., MacRae C., Silveira de Carvalho L.R. (2024). Standardizing CRISPR-Cas13 knockdown technique to investigate the role of cdh2 gene in pituitary development through growth hormone expression and transcription factors. Front. Endocrinol..

[144] Dong Z., Ge J., Xu Z., Dong X., Cao S., Pan J., Zhao Q. (2014). Generation of myostatin B knockout yellow catfish (*Tachysurus fulvidraco*) using transcription activator-like effector nucleases. Zebrafish.

[145] Qin G., Qin Z., Lu C., Ye Z., Elaswad A., Bangs M., Li H., Zhang Y., Huang Y., Shi H. (2022). Gene editing of the catfish gonadotropin-releasing hormone gene and hormone therapy to control the reproduction in channel catfish, *Ictalurus punctatus*. Biology.

[146] Moore F.E., Reyon D., Sander J.D., Martinez S.A., Blackburn J.S., Khayter C., Ramirez C.L., Joung J.K., Langenau D.M. (2012). Improved somatic mutagenesis in zebrafish using transcription activator-like effector nucleases (TALENs). PLoS ONE.

[147] Ansai S., Sakuma T., Yamamoto T., Ariga H., Uemura N., Takahashi R., Kinoshita M. (2013). Efficient targeted mutagenesis in medaka using custom-designed transcription activator-like effector nucleases. Genetics.

[148] Qin Z., Li Y., Su B., Cheng Q., Ye Z., Perera D.A., Fobes M., Shang M., Dunham R.A. (2016). Editing of the luteinizing hormone gene to sterilize channel catfish, *Ictalurus punctatus*, using a modified zinc finger nuclease technology with electroporation. Mar. Biotechnol..

[149] Dong Z., Ge J., Li K., Xu Z., Liang D., Li J., Li J., Jia W., Li Y., Dong X. (2011). Heritable targeted inactivation of myostatin gene in yellow catfish (*Pelteobagrus fulvidraco*) using engineered zinc finger nucleases. PLoS ONE.

[150] Doyon Y., McCammon J.M., Miller J.C., Faraji F., Ngo C., Katibah G.E., Amora R., Hocking T.D., Zhang L., Rebar E.J. (2008). Heritable targeted gene disruption in zebrafish using designed zinc-finger nucleases. Nat. Biotechnol..

[151] Ansai S., Ochiai H., Kanie Y., Kamei Y., Gou Y., Kitano T., Yamamoto T., Kinoshita M. (2012). Targeted disruption of exogenous EGFP gene in medaka using zinc-finger nucleases. Dev. Growth Differ..

[152] Foley J.E., Maeder M.L., Pearlberg J., Joung J.K., Peterson R.T., Yeh J.-R.J. (2009). Targeted mutagenesis in zebrafish using customized zinc-finger nucleases. Nat. Protoc..

[153] Guan G., Zhang X., Naruse K., Nagahama Y., Hong Y. (2014). Gene replacement by zinc finger nucleases in medaka embryos. Mar. Biotechnol..

[154] Zhong Z., Niu P., Wang M., Huang G., Xu S., Sun Y., Xu X., Hou Y., Sun X., Yan Y. (2016). Targeted disruption of *sp7* and myostatin with CRISPR-Cas9 results in severe bone defects and more muscular cells in common carp. Sci. Rep..

[155] Li M., Feng R., Ma H., Dong R., Liu Z., Jiang W., Tao W., Wang D. (2016). Retinoic acid triggers meiosis initiation via *stra8*-dependent pathway in southern catfish, *Silurus meridionalis*. Gen. Comp. Endocrinol..

[156] Qin G., Qin Z., Lu C., Ye Z., Elaswad A., Jin Y., Khan M.G.Q., Su B., Dunham R.A. (2023). Gene editing of the follicle-stimulating hormone gene to sterilize channel catfish, *Ictalurus punctatus*, using a modified transcription activator-like effector nuclease technology with electroporation. Biology.

[157] Coogan M., Alston V., Su B., Khalil K., Elaswad A., Khan M., Simora R.M., Johnson A., Xing D., Li S. (2022). CRISPR/Cas-9 induced knockout of myostatin gene improves growth and disease resistance in channel catfish (*Ictalurus punctatus*). Aquaculture.

[158] Elaswad A., Khalil K., Ye Z., Liu Z., Liu S., Peatman E., Odin R., Vo K., Drescher D., Gosh K. (2018). Effects of CRISPR/Cas9 dosage on TICAM1 and RBL gene mutation rate, embryonic development, hatchability and fry survival in channel catfish. Sci. Rep..

[159] Xing D., Su B., Bangs M., Li S., Wang J., Bern L., Simora R.M.C., Wang W., Ma X., Coogan M. (2022). CRISPR/Cas9-mediated knock-in method can improve the expression and effect of transgene in P1 generation of channel catfish (*Ictalurus punctatus*). Aquaculture.

[160] Xing D., Li S., Shang M., Wang W., Zhang Q., Wang J., Hasin T., Hettiarachchi D., Alston V., Bern L. (2022). A new strategy for increasing knock-in efficiency: Multiple elongase and desaturase transgenes knock-in by targeting long repeated sequences. ACS Synth. Biol..

[161] Wang J., Su B., Bruce T.J., Wise A.L., Zeng P., Cao G., Simora R.M.C., Bern L., Shang M., Li S. (2023). CRISPR/Cas9 microinjection of transgenic embryos enhances the dual-gene integration efficiency of antimicrobial peptide genes for bacterial resistance in channel catfish, *Ictalurus punctatus*. Aquaculture.

[162] Booncherd K., Sreebun S., Pasomboon P., Boonanuntanasarn S. (2024). Effects of CRISPR/Cas9-mediated *dnd1* knockout impairs gonadal development in striped catfish. Animal.

[163] Kishimoto K., Washio Y., Yoshiura Y., Toyoda A., Ueno T., Fukuyama H., Kato K., Kinoshita M. (2018). Production of a breed of red sea bream *Pagrus major* with an increase of skeletal muscle mass and reduced body length by genome editing with CRISPR/Cas9. Aquaculture.

[164] Kim J., Cho J.Y., Kim J.-W., Kim H.-C., Noh J.K., Kim Y.-O., Hwang H.-K., Kim W.-J., Yeo S.-Y., An C.M. (2019). CRISPR/Cas9-mediated myostatin disruption enhances muscle mass in the olive flounder *Paralichthys olivaceus*. Aquaculture.

[165] Zheng J., Liu S., Jiang W., Li F., Chi M., Cheng S., Liu Y. (2023). CRISPR/Cas9-mediated mutation of *mstn* confers growth performance in *Culter alburnus* juveniles. Aquac. Fish..

[166] Song Y., Cone R.D. (2007). Creation of a genetic model of obesity in a teleost. FASEB J..

[167] Kobayashi Y., Tsuchiya K., Yamanome T., Schiöth H.B., Kawauchi H., Takahashi A. (2008). Food deprivation increases the expression of melanocortin-4 receptor in the liver of barfin flounder, *Verasper moseri*. Gen. Comp. Endocrinol..

[168] Schjolden J., Schiöth H.B., Larhammar D., Winberg S., Larson E.T. (2009). Melanocortin peptides affect the motivation to feed in rainbow trout (*Oncorhynchus mykiss*). Gen. Comp. Endocrinol..

[169] Yang Y., Li Q., Shu H., Zhou H., Li X., Hou L. (2017). Characterization of the melanocortin-4 receptor gene from *Spinibarbus hollandi* and the association between its polymorphisms and *S. hollandi* growth traits. Fish. Sci..

[170] Liu R., Kinoshita M., Adolfi M.C., Schartl M. (2019). Analysis of the role of the Mc4r system in development, growth, and puberty of medaka. Front. Endocrinol..

[171] Weide K., Christ N., Moar K.M., Arens J., Hinney A., Mercer J.G., Eiden S., Schmidt I. (2003). Hyperphagia, not hypometabolism, causes early onset obesity in melanocortin-4 receptor knockout mice. Physiol. Genom..

[172] Fei F., Sun S.-y., Yao Y.-x., Wang X. (2017). Generation and phenotype analysis of zebrafish mutations of obesity-related genes *lepr* and *mc4r*. Acta Physiol. Sin..

[173] Dunham R.A., Winn R.N., Pinkert C.A. (2014). Production of transgenic fish. Transgenic Animal Technology.

[174] Ma J., Fan Y., Zhou Y., Liu W., Jiang N., Zhang J., Zeng L. (2018). Efficient resistance to grass carp reovirus infection in JAM-A knockout cells using CRISPR/Cas9. Fish Shellfish Immunol..

[175] Dehler C.E., Lester K., Della Pelle G., Jouneau L., Houel A., Collins C., Dovgan T., Machat R., Zou J., Boudinot P. (2019). Viral resistance and IFN signaling in STAT2 knockout fish cells. J. Immunol..

[176] Zhu J., Liu X., Cai X., Ouyang G., Fan S., Wang J., Xiao W. (2020). Zebrafish *prmt7* negatively regulates antiviral responses by suppressing the retinoic acid-inducible gene-I-like receptor signaling. FASEB J..

[177] Beck B.H., Farmer B.D., Straus D.L., Li C., Peatman E. (2012). Putative roles for a rhamnose binding lectin in *Flavobacterium columnare* pathogenesis in channel catfish *Ictalurus punctatus*. Fish Shellfish Immunol..

[178] Baoprasertkul P., Peatman E., Somridhivej B., Liu Z. (2006). Toll-like receptor 3 and TICAM genes in catfish: Species-specific expression profiles following infection with *Edwardsiella ictaluri*. Immunogenetics.

[179] Barksdale S.M., Hrifko E.J., van Hoek M.L. (2017). Cathelicidin antimicrobial peptide from *Alligator mississippiensis* has antibacterial activity against multi-drug resistant *Acinetobacter baumanii* and *Klebsiella pneumoniae*. Dev. Comp. Immunol..

[180] Wang J., Torres I.M., Shang M., Al-Armanazi J., Dilawar H., Hettiarachchi D.U., Paladines-Parrales A., Chambers B., Pottle K., Soman M. (2024). Direct and pleiotropic effects of antimicrobial peptide transgene integration on reproductive, growth regulating, and non-coding loci in channel catfish (*Ictalurus punctatus*). Agric. Commun..

[181] Nie C.H., Hilsdorf A.W., Wan S.M., Gao Z.X. (2020). Understanding the development of intermuscular bones in teleost: Status and future directions for aquaculture. Rev. Aquacult..

[182] Xu X., Zheng J., Qian Y., Luo C. (2015). Normally grown and developed intermuscular bone-deficient mutant in grass carp, *Ctenopharyngodon idellus*. Chin. Sci. Bull..

[183] Xu H., Tong G., Yan T., Dong L., Yang X., Dou D., Sun Z., Liu T., Zheng X., Yang J. (2022). Transcriptomic analysis provides insights to reveal the bmp6 function related to the development of intermuscular bones in zebrafish. Front. Cell Dev. Biol..

[184] Dong Q., Nie C.-H., Wu Y.-M., Zhang D.-Y., Wang X.-D., Tu T., Jin J., Tian Z.-Y., Liu J.-Q., Xiao Z.-Y. (2023). Generation of blunt snout bream without intermuscular bones by *runx2b* gene mutation. Aquaculture.

[185] Gan R.-H., Li Z., Wang Z.-W., Li X.-Y., Wang Y., Zhang X.-J., Tong J.-F., Wu Y., Xia L.-Y., Gao Z.-X. (2023). Creation of intermuscular bone-free mutants in amphitriploid gibel carp by editing two duplicated *runx2b* homeologs. Aquaculture.

[186] Datsomor A.K., Zic N., Li K., Olsen R.E., Jin Y., Vik J.O., Edvardsen R.B., Grammes F., Wargelius A., Winge P. (2019). CRISPR/Cas9-mediated ablation of elovl2 in Atlantic salmon (*Salmo salar* L.) inhibits elongation of polyunsaturated fatty acids and induces Srebp-1 and target genes. Sci. Rep..

[187] Datsomor A.K., Olsen R.E., Zic N., Madaro A., Bones A.M., Edvardsen R.B., Wargelius A., Winge P. (2019). CRISPR/Cas9-mediated editing of Δ5 and Δ6 desaturases impairs Δ8-desaturation and docosahexaenoic acid synthesis in Atlantic salmon (*Salmo salar* L.). Sci. Rep..

[188] Lo J.H., Lin C.-M., Chen M.J., Chen T.T. (2014). Altered gene expression patterns of innate and adaptive immunity pathways in transgenic rainbow trout harboring cecropin P1 transgene. BMC Genom..

[189] Su B., Shang M., Li C., Perera D.A., Pinkert C.A., Irwin M.H., Peatman E., Grewe P., Patil J.G., Dunham R.A. (2015). Effects of transgenic sterilization constructs and their repressor compounds on hatch, developmental rate and early survival of electroporated channel catfish embryos and fry. Transgenic Res..

[190] Han Y.-C., Lin C.-M., Chen T.T. (2018). RNA-Seq analysis of differentially expressed genes relevant to innate and adaptive immunity in cecropin P1 transgenic rainbow trout (*Oncorhynchus mykiss*). BMC Genom..

[191] Wang W.-B., Wang Y.-P., Hu W., Li A.-H., Cai T.-Z., Zhu Z.-Y., Wang J.-G. (2006). Effects of the “all-fish” growth hormone transgene expression on non-specific immune functions of common carp, *Cyprinus carpio* L.. Aquaculture.

[192] Ling F., Luo Q., Wang J.-G., Wang Y.-P., Wang W.-B., Gong X.-N. (2009). Effects of the “all-fish” GH (growth hormone) transgene expression on resistance to *Ichthyophthirius multifiliis* infections in common carp, *Cyprinus carpio* L.. Aquaculture.

[193] Jhingan E., Devlin R., Iwama G. (2003). Disease resistance, stress response and effects of triploidy in growth hormone transgenic coho salmon. J. Fish Biol..

[194] Sundström L.F., Lohmus M., Johnsson J.I., Devlin R.H. (2004). Growth hormone transgenic salmon pay for growth potential with increased predation mortality. Proc. R. Soc. Lond. Ser. B Biol. Sci..

[195] Dunham R.A., Chitmanat C., Nichols A., Argue B., Powers D.A., Chen T.T. (1999). Predator avoidance of transgenic channel catfish containing salmonid growth hormone genes. Mar. Biotechnol..

[196] Dunham R.A. (2011). Aquaculture and Fisheries Biotechnology: Genetic Approaches.

[197] Abass N.Y., Elwakil H.E., Hemeida A.A., Abdelsalam N.R., Ye Z., Su B., Alsaqufi A.S., Weng C.-C., Trudeau V.L., Dunham R.A. (2016). Genotype–environment interactions for survival at low and sub-zero temperatures at varying salinity for channel catfish, hybrid catfish and transgenic channel catfish. Aquaculture.

[198] Abass N.Y., Drescher D., Backenstose N., Ye Z., Vo K., Odin R., Qin G., Dong S., Dunham R.A. (2020). Genotype-environment interactions for survival and growth rate at varying levels of sodium chloride for growth hormone transgenic channel catfish (*Ictalurus punctatus*), channel catfish, and albino channel catfish. Aquaculture.

[199] Chiang Y.-A., Kinoshita M., Maekawa S., Kulkarni A., Lo C.-F., Yoshiura Y., Wang H.-C., Aoki T. (2016). TALENs-mediated gene disruption of myostatin produces a larger phenotype of medaka with an apparently compromised immune system. Fish Shellfish Immunol..

[200] Chisada S.-I., Okamoto H., Taniguchi Y., Kimori Y., Toyoda A., Sakaki Y., Takeda S., Yoshiura Y. (2011). Myostatin-deficient medaka exhibit a double-muscling phenotype with hyperplasia and hypertrophy, which occur sequentially during post-hatch development. Dev. Biol..

[201] Lien S., Koop B.F., Sandve S.R., Miller J.R., Kent M.P., Nome T., Hvidsten T.R., Leong J.S., Minkley D.R., Zimin A. (2016). The Atlantic salmon genome provides insights into rediploidization. Nature.

[202] Kosicki M., Tomberg K., Bradley A. (2018). Repair of double-strand breaks induced by CRISPR–Cas9 leads to large deletions and complex rearrangements. Nat. Biotechnol..

[203] Park S.H., Cao M., Pan Y., Davis T.H., Saxena L., Deshmukh H., Fu Y., Treangen T., Sheehan V.A., Bao G. (2022). Comprehensive analysis and accurate quantification of unintended large gene modifications induced by CRISPR-Cas9 gene editing. Sci. Adv..

[204] Okoli A.S., Blix T., Myhr A.I., Xu W., Xu X. (2022). Sustainable use of CRISPR/Cas in fish aquaculture: The biosafety perspective. Transgenic Res..

[205] Farrell A.P., Bennett W., Devlin R.H. (1997). Growth-enhanced transgenic salmon can be inferior swimmers. Can. J. Zool..

[206] Dunham R., Chen T., Powers D., Nichols A., Argue B., Chitmanat C. (1995). Predator avoidance, spawning, and foraging ability of transgenic channel catfish with rainbow trout growth hormone gene. Biotechnology Risk Assessment, Proceedings of the Biotechnology Risk Assessment Symposium, Reston, VA, USA, 6–8 June 1995.

[207] Ledesma A.V., Van Eenennaam A.L. (2024). Global status of gene edited animals for agricultural applications. Vet. J..

[208] Kondo K., Taguchi C. (2022). Japanese regulatory framework and approach for genome-edited foods based on latest scientific findings. Food Saf..

[209] Hjort C., Cole J., Frébort I. (2021). European genome editing regulations: Threats to the European bioeconomy and unfit for purpose. EFB Bioecon. J..

[210] Wolters W.R., Lilyestrom C.G., Craig R.J. (1991). Growth, yield, and dress-out percentage of diploid and triploid channel catfish in earthen ponds. Progress. Fish-Cult..

[211] Chrisman C.L., Wolters W.R., Libey G.S. (1983). Triploidy in channel catfish. J. World Maric. Soc..

[212] Lilyestrom C., Wolters W., Bury D., Rezk M., Dunham R. (1999). Growth, carcass traits, and oxygen tolerance of diploid and triploid catfish hybrids. N. Am. J. Aquac..

[213] Li H., Su B., Qin G., Ye Z., Alsaqufi A., Perera D.A., Shang M., Odin R., Vo K., Drescher D. (2017). Salt sensitive tet-off-like systems to knockdown primordial germ cell genes for repressible transgenic sterilization in channel catfish, *Ictalurus punctatus*. Mar. Drugs.

[214] Li H., Su B., Qin G., Ye Z., Elaswad A., Alsaqufi A., Perera D.A., Qin Z., Odin R., Vo K. (2018). Repressible transgenic sterilization in channel catfish, *Ictalurus punctatus*, by knockdown of primordial germ cell genes with copper-sensitive constructs. Mar. Biotechnol..

[215] Simora R.M.C. (2020). Transgene Insertion of Cathelicidin Gene in Channel Catfish *Ictalurus punctatus* Using CRISPR/Cas9 Knock-in Technology and Cathelicidin Activity Against Catfish Pathogens. Ph.D. Thesis.

[216] Mussolino C., Alzubi J., Fine E.J., Morbitzer R., Cradick T.J., Lahaye T., Bao G., Cathomen T. (2014). TALENs facilitate targeted genome editing in human cells with high specificity and low cytotoxicity. Nucleic Acids Res..

[217] Gaj T., Gersbach C.A., Barbas C.F. (2013). ZFN, TALEN, and CRISPR/Cas-based methods for genome engineering. Trends Biotechnol..

[218] Blix T.B., Dalmo R.A., Wargelius A., Myhr A.I. (2021). Genome editing on finfish: Current status and implications for sustainability. Rev. Aquacult..

[219] Marvel M., Spicer O.S., Wong T.-T., Zmora N., Zohar Y. (2018). Knockout of the Gnrh genes in zebrafish: Effects on reproduction and potential compensation by reproductive and feeding-related neuropeptides. Biol. Reprod..

[220] Porto E.M., Komor A.C., Slaymaker I.M., Yeo G.W. (2020). Base editing: Advances and therapeutic opportunities. Nat. Rev. Drug Discov..

[221] Basharat R., Rizzo G., Zoodsma J.D., Wollmuth L.P., Sirotkin H.I. (2025). Optimizing prime editing in zebrafish. Cris. J..

[222] Wessels H.-H., Stirn A., Méndez-Mancilla A., Kim E.J., Hart S.K., Knowles D.A., Sanjana N.E. (2024). Prediction of on-target and off-target activity of CRISPR–Cas13d guide RNAs using deep learning. Nat. Biotechnol..

[223] Kamran M., Zhijun T., Ullah A., Alvi M.H.A., Aqeel T., Xuejun C., Sagheer S., Bingguang X., Xu H. (2026). Integrating genomic technologies with artificial intelligence, CRISPR editing, and high-throughput phenotyping for engineering disease-resistant crops. Plant Stress.

[224] Liu Y., Zhu K., Peng W., Liu Z., Mao X. (2026). Multi-omics and artificial intelligence for precision drug discovery and potential clinical applications. Signal Transduct. Target. Ther..

[225] Luo Z., Yu Y., Bao Z., Li F. (2024). Evaluation of machine learning method in genomic selection for growth traits of Pacific white shrimp. Aquaculture.

[226] Dunham R.A., Majumdar K., Hallerman E., Bartley D., Mair G., Hulata G., Liu Z., Pongthana N., Bakos J., Penman D., Subasinghe R.P., Bueno P., Phillips M.J., Hough C., McGladdery S.E., Arthur J.R. (2000). Review of the Status of Aquaculture Genetics. Aquaculture in the Third Millennium, Technical Proceedings of the Conference on Aquaculture in the Third Millennium, Bangkok, Thailand, 10–25 February 2000.

[227] Sgroi F., Musso D. (2022). Urban food strategies and sustainable agri-food systems: Results of empirical analysis in Palermo. J. Agric. Food Res..

[228] Chapman R., Likhanov M., Selita F., Zakharov I., Smith-Woolley E., Kovas Y. (2019). New literacy challenge for the twenty-first century: Genetic knowledge is poor even among well educated. J. Community Genet..

[229] Cummings C., Selfa T., Lindberg S., Bain C. (2024). Identifying public trust building priorities of gene editing in agriculture and food. Agric. Hum. Values.

[230] Siegrist M. (2000). The influence of trust and perceptions of risks and benefits on the acceptance of gene technology. Risk Anal..

[231] Lindberg S.A., Peters D.J., Cummings C.L. (2023). Gene-edited food adoption intentions and institutional trust in the United States: Benefits, acceptance, and labeling. Rural Sociol..

[232] Tenbült P., de Vries N.K., Dreezens E., Martijn C. (2005). Perceived naturalness and acceptance of genetically modified food. Appetite.

[233] Robinson N.A., Østbye T.K.K., Kettunen A.H., Coates A., Barrett L.T., Robledo D., Dempster T. (2024). A guide to assess the use of gene editing in aquaculture. Rev. Aquacult..

